# A second wave of *Salmonella* T3SS1 activity prolongs the lifespan of infected epithelial cells

**DOI:** 10.1371/journal.ppat.1006354

**Published:** 2017-04-20

**Authors:** Ciaran E. Finn, Audrey Chong, Kendal G. Cooper, Tregei Starr, Olivia Steele-Mortimer

**Affiliations:** Laboratory of Bacteriology, Rocky Mountain Laboratories, National Institutes of Allergy and Infectious Diseases, National Institutes of Health, Hamilton, Montana, United States of America; Stanford University School of Medicine, UNITED STATES

## Abstract

Type III secretion system 1 (T3SS1) is used by the enteropathogen *Salmonella enterica* serovar Typhimurium to establish infection in the gut. Effector proteins translocated by this system across the plasma membrane facilitate invasion of intestinal epithelial cells. One such effector, the inositol phosphatase SopB, contributes to invasion and mediates activation of the pro-survival kinase Akt. Following internalization, some bacteria escape from the *Salmonella*-containing vacuole into the cytosol and there is evidence suggesting that T3SS1 is expressed in this subpopulation. Here, we investigated the post-invasion role of T3SS1, using SopB as a model effector. In cultured epithelial cells, SopB-dependent Akt phosphorylation was observed at two distinct stages of infection: during and immediately after invasion, and later during peak cytosolic replication. Single cell analysis revealed that cytosolic *Salmonella* deliver SopB via T3SS1. Although intracellular replication was unaffected in a SopB deletion mutant, cells infected with Δ*sopB* demonstrated a lack of Akt phosphorylation, earlier time to death, and increased lysis. When SopB expression was induced specifically in cytosolic *Salmonella*, these effects were restored to levels observed in WT infected cells, indicating that the second wave of SopB protects this infected population against cell death via Akt activation. Thus, T3SS1 has two, temporally distinct roles during epithelial cell colonization. Additionally, we found that delivery of SopB by cytosolic bacteria was translocon-independent, in contrast to canonical effector translocation across eukaryotic membranes, which requires formation of a translocon pore. This mechanism was also observed for another T3SS1 effector, SipA. These findings reveal the functional and mechanistic adaptability of a T3SS that can be harnessed in different microenvironments.

## Author summary

Non-Typhoidal *Salmonella* are important agents of food borne disease worldwide. These facultative intracellular bacteria use a specialized Type III Secretion (T3SS1) system to invade intestinal epithelial cells. Effector proteins translocated by this system across the eukaryotic plasma membrane induce actin rearrangements and target signaling pathways. One such effector is SopB, which contributes to invasion and mediates activation of the pro-survival kinase Akt. Within epithelial cells, *Salmonella* survive and replicate within a modified phagosome, known as the *Salmonella*-containing vacuole, or the host cell cytosol. Here, we investigated the post-invasion role of T3SS1 in epithelial cells, using SopB as a model effector. SopB-dependent Akt phosphorylation was observed at two distinct stages of infection: during and immediately after invasion, and later during peak cytosolic replication. SopB delivery by cytosolic *Salmonella* required T3SS1 but was translocon-independent. This was also observed for another T3SS1 effector, SipA, indicating that T3SS1 effectors may be secreted directly into the cytosol. Infection with a SopB deletion mutant eliminated the induction of Akt phosphorylation and decreased the lifespan of infected cells. These effects were reversed by expressing SopB specifically in cytosolic bacteria, confirming a role for SopB and T3SS1 during the cytosolic stage of infection. Thus, T3SS1 has two temporally distinct roles during epithelial cell colonization.

## Introduction

Type III Secretion Systems (T3SSs) are used by a variety of Gram-negative bacteria for interkingdom delivery of proteins (known as effectors) from the bacterial cytosol into eukaryotic cells [1]. For bacterial pathogens, such as *Salmonella enterica*, *Yersinia* spp, and pathogenic *Escherichia coli*, these molecular syringes are key virulence determinants essential for a variety of processes including: adherence; invasion; intracellular survival and cytotoxicity. This broad repertoire is due to the diversified nature of effectors rather than the mechanism of delivery, which is highly conserved [2,3]. T3SS delivery is a contact-dependent process characterized by the formation of a pore, or translocon, at the point of contact with the eukaryotic membrane and through which effectors are delivered into the host cell [4].

*Salmonella enterica* serovar Typhimurium (hereafter *Salmonella*), a leading cause of gastroenteritis, possesses two functionally distinct T3SSs, encoded on *Salmonella* Pathogenicity Islands 1 and 2 (SPI1 and SPI2) [5]. The SPI1-encoded T3SS1 triggers invasion of non-phagocytic cells, such as intestinal epithelial cells, following contact with the plasma membrane. A cohort of translocated effectors targets the actin network and membrane phospholipids to direct formation of membrane ruffles, leading to uptake of the bacterium into a modified phagosome known as the *Salmonella* Containing Vacuole (SCV) [6]. Within the SCV, the SPI1 regulon is rapidly down-regulated whereas the SPI2 regulon is induced. Consequently, SCV biogenesis is primarily determined by effectors translocated via T3SS2 [7].

In epithelial cells, some *Salmonella* escape from the SCV and can survive and replicate in the cytosol resulting in two distinct populations of intracellular bacteria [8,9]. Cytosolic *Salmonella* replicate faster than vacuolar bacteria [9,10] and this “hyper-replication” results in a subpopulation of infected cells that are filled with *Salmonella*, both *in vitro* and *in vivo* [8]. In cell culture models, such as HeLa and C2BBe1 cells, cytosolic replication occurs in a largely synchronous fashion starting at ~4 h post-infection (hpi) and continuing for several hours until the inflammasome mediated death of the host cell at ~8–10 hpi [8,9,11,12]. In contrast, the contribution from vacuolar bacteria to intracellular replication is primarily seen from 12 hpi onwards [9]. Thus, within a time frame of 4–10 hpi, cells containing cytosolic *Salmonella* can be distinguished from those containing only vacuolar *Salmonella* due to the higher bacterial numbers. Additionally, these populations can be differentiated using fluorescent transcriptional reporters for SPI1 and SPI2 genes during this time period. SPI2-induced bacteria are only observed in the vacuole whereas SPI1 induction has only been observed in cytosolic hyper-replicating bacteria [8,12].

Several lines of evidence suggest that the SPI1-encoded T3SS1 has post-invasion activities in addition to its well characterized role in invasion. For example, the T3SS1 effector SopB (SigD), contributes to actin remodeling and induces phosphorylation of the pro-survival kinase Akt during invasion of epithelial cells [13–16], but SopB-dependent Akt phosphorylation can be detected for several hours following invasion [15] and it has been implicated in intracellular replication of *Salmonella* [17]. It is not clear whether the late activity of SopB can be attributed solely to effector translocated by T3SS1 during or post-invasion, or if it can be translocated by T3SS2. An alternative possibility is that the SP1-induced cytosolic subpopulation of intracellular *Salmonella* delivers *de novo* synthesized SopB. However, it remains to be determined whether T3SS1 is functionally active in this intracellular population of bacteria.

Here, we investigated the post-invasion intracellular role of T3SS1 using SopB as a model effector and Akt phosphorylation as an indicator of effector activity. Our study reveals widespread expression and activity of T3SS1 in cytosolic *Salmonella*. This activity results in delivery of a second wave of SopB, leading to resurgence in Akt phosphorylation and ultimately prolonging the lifespan of this subpopulation of infected cells. Unexpectedly, effector delivery was not inhibited in the absence of the translocon, indicating that effector delivery by cytosolic *Salmonella* can occur via a non-canonical translocon-independent mechanism.

## Results

### Akt phosphorylation is sustained in epithelial cells containing cytosolic hyper-replicating *Salmonella*

**Fig 1:**
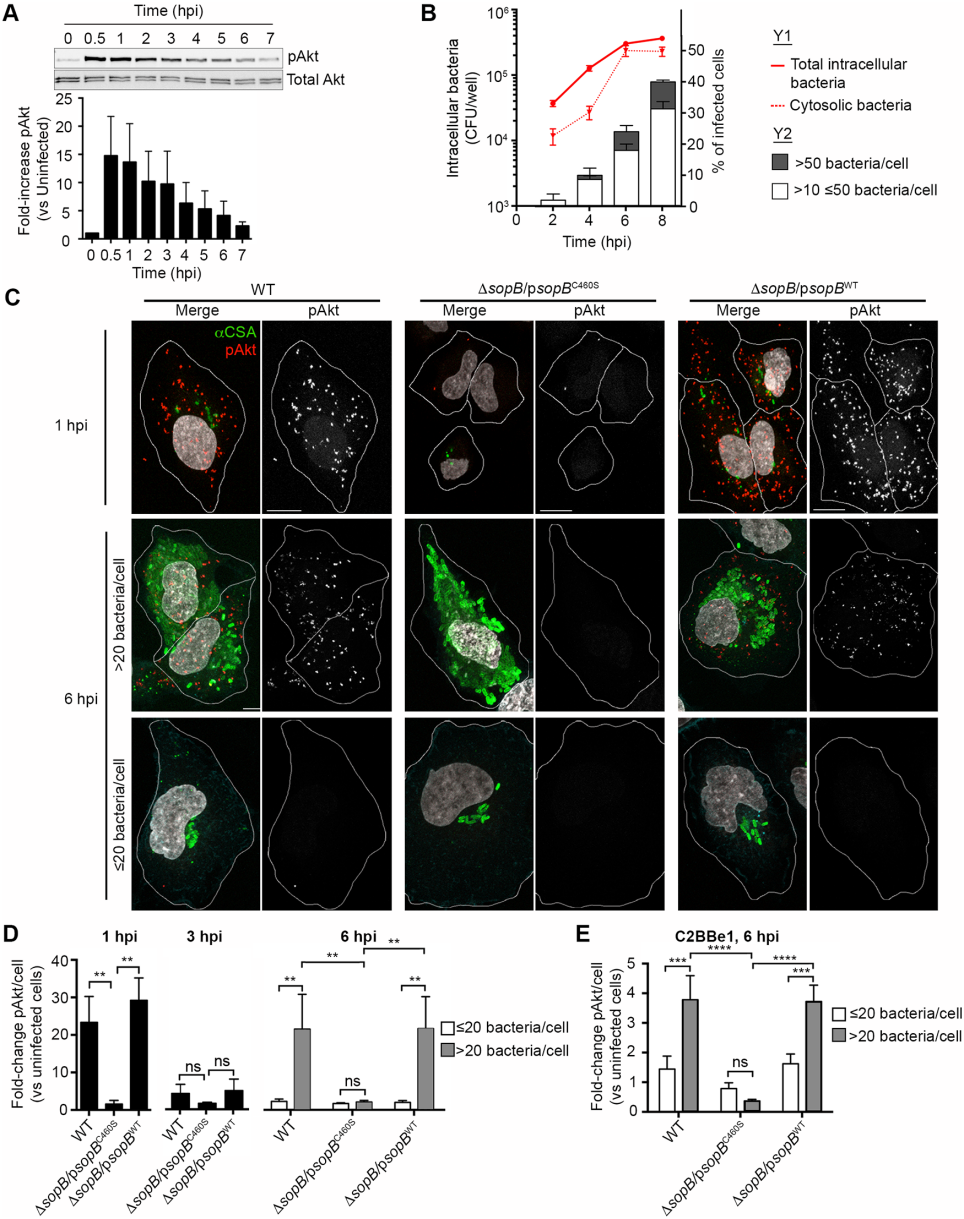
Akt phosphorylation occurs in two distinct phases in *Salmonella*-infected epithelial cells. HeLa (A-D) or C2BBe1 (E) cells were infected with *Salmonella* Typhimurium. (A) Akt phosphorylation in infected HeLa cells peaks within the first 2 h following invasion but persists at a low level for 6–7 hpi. Western blot for phosphorylated Akt (pAkt) and total Akt, and band densitometry, normalized to total Akt (A) and uninfected (D and E), for phosphorylated Akt. Densitometry data show means ± SD for 3 independent experiments. Western blot is from one representative experiment. (B) Cytosolic replication of *Salmonella* is pronounced at 6–8 hpi and is characterized by the appearance of cells containing > 50 bacteria. Intracellular CFU were assessed by gentamicin protection assay, with (cytosolic bacteria, dotted red line) or without (total intracellular bacteria, solid red line) the addition of chloroquine. For analysis of intracellular bacteria in individual cells (Y2), cells were fixed and processed for immunostaining (bacteria) and intracellular bacteria counted by immunofluorescence microscopy. (C-E) Akt phosphorylation in individual cells. *Salmonella*-infected cells were fixed and processed for immunostaining (bacteria) and proximity ligation assay (PLA, pAkt). DNA was stained with DAPI. (C) Representative confocal images of infected HeLa cells. Scale bars: 10 μm. Analysis of pAkt levels in HeLa (D) or C2BBe1 (E) cells containing 1–20 bacteria/cell or >20 bacteria/cell. The pAkt spots/cell were counted and normalized to uninfected cells. Means ± SD of 3 independent experiments. ** P ≤ 0.01, *** P ≤ 0.001.

We previously reported that the T3SS1 effector SopB induces sustained Akt phosphorylation following T3SS1-mediated invasion of epithelial cells by *Salmonella* Typhimurium [14,15]. Akt phosphorylation is readily detected in infected HeLa cell lysates using antibodies that specifically recognize phosphorylated Akt. Although Akt phosphorylation peaks at 0.5–1 hpi, a low level of phosphorylation is sustained for at least 7 hpi (Fig 1A). We hypothesized that this persistent Akt phosphorylation could be due to either: a low level of phosphorylation in all infected cells; or a high level of phosphorylation in a subpopulation of infected cells. In particular, we wondered if Akt phosphorylation levels were different in cells containing cytosolic vs vacuolar *Salmonella*. These two distinct intracellular populations can be resolved in infected HeLa cells by a modified gentamicin protection assay, in which chloroquine is used to selectively kill vacuolar bacteria, or by fluorescence microscopy (Fig 1B). We focused these studies on the time period from 2–8 hpi, which encompasses the peak of cytosolic replication [9,12]. In order to synchronize invasion, and the subsequent intracellular processes, we grew the *Salmonella* under SPI1-inducing conditions and then used a high moi (approximately 50 bacteria/cell) and a relatively short infection time (10 min) as previously described [18,19]. Under these conditions, the number of bacteria internalized into each cell is controlled so that over 95% of infected cells contain 1–10 bacteria at 2 hpi (Fig 1B). Following the onset of intracellular replication, the number of bacteria per cell increases and by 8 hpi approximately 40% of infected cells contain >10 bacteria. Approximately 25% of these cells, or 10% of total infected cells, contain hyper-replicating cytosolic bacteria and can be readily identified due to the high number (>50 per cell) of intracellular bacteria [8,9,12]. To determine whether Akt phosphorylation is increased in cells containing cytosolic bacteria, we used fluorescence microscopy combined with *in situ* proximity ligation amplification (PLA), a sensitive and specific method to detect low levels of proteins [20]. In cells infected with WT *Salmonella*, confocal microscopy revealed high levels of phosphorylated Akt (pAkt) compared to uninfected cells (Fig 1C and 1D). At 1 hpi the pAkt signal was 20- to 30-fold higher in cells containing WT *Salmonella* compared to uninfected cells in the same monolayer (Fig 1D). Akt phosphorylation was similarly increased in cells infected with a SopB deletion mutant (Δ*sopB*) complemented with plasmid borne SopB (p*sopB*^WT^) but not a catalytically inactive mutant (p*sopB*^C460S^) [14,15]. This SopB-dependent Akt phosphorylation was transient since, by 3 hpi, pAkt levels in infected cells decreased to near background levels and were not significantly affected by the presence or absence of active SopB (Fig 1D). A resurgence of Akt phosphorylation was observed at 6 hpi, although only in a subset of infected cells. We observed a clear correlation between the number of intracellular bacteria and levels of pAkt, which was 20- to 30-fold higher than background in cells containing ≥20 bacteria but was at background levels in cells containing <20 bacteria (Fig 1C and 1D). This cut off was selected as we have previously shown that, within this time frame, cells with 20 or more bacteria contained predominantly cytosolic *Salmonella* [8]. As at 1 hpi the increase in pAkt was dependent on the catalytic activity of SopB (Fig 1C and 1D). Similar results were seen in C2BBe1 colorectal epithelial cells (Fig 1E). These results show that, at a single cell level, there are two distinct periods of SopB-dependent Akt phosphorylation. The first phase is widely induced during invasion and is largely depleted by 3 hpi, whereas the second phase is induced later (observed at 6 hpi) in the subpopulation of infected cells that contain greater than 20, likely cytosolic, bacteria.

### The T3SS1 effector, SopB, is present in cells containing hyper-replicating cytosolic *Salmonella*

**Fig 2:**
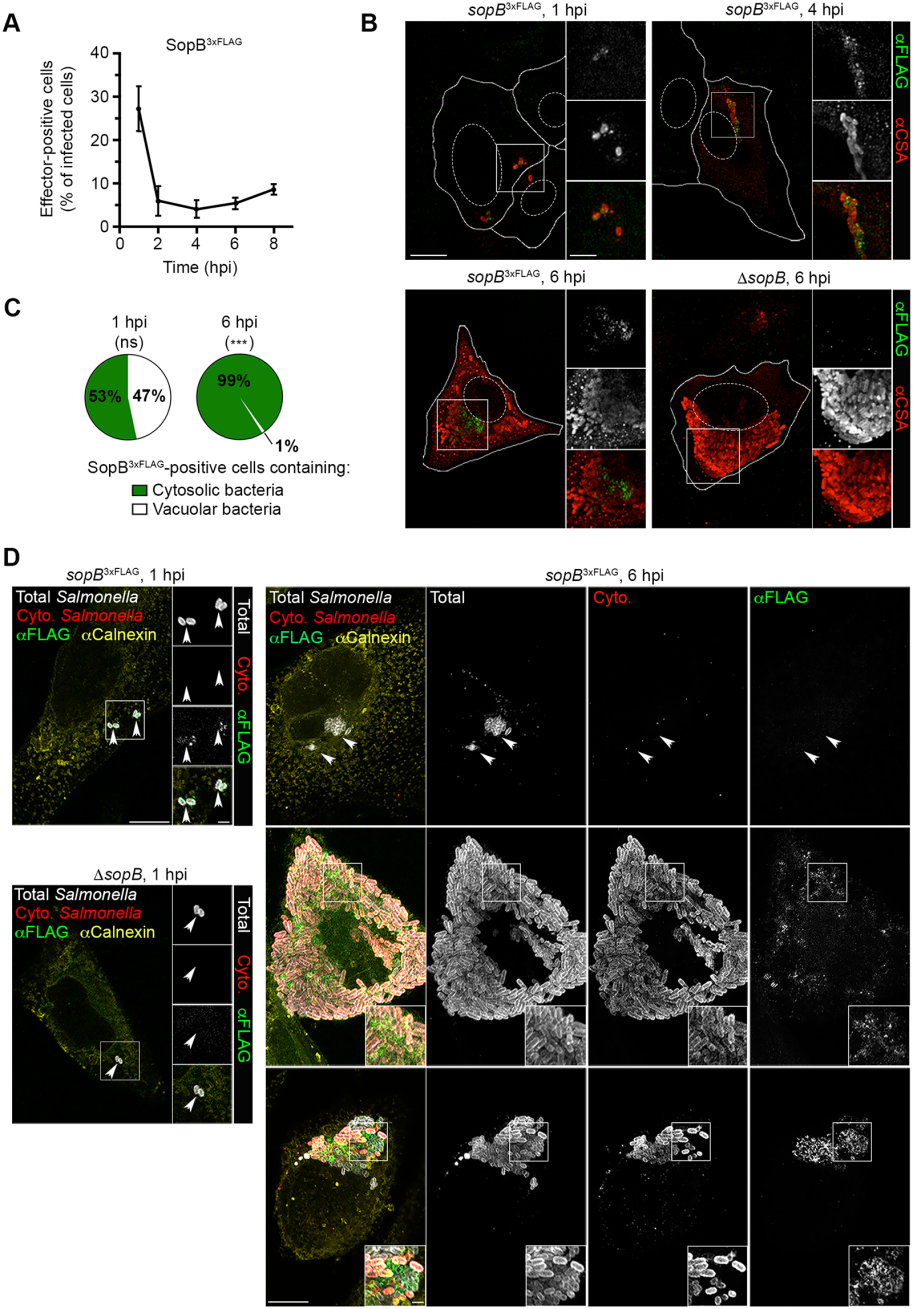
Sustained delivery of SopB into epithelial cells by *Salmonella*. Time course of SopB delivery in HeLa cells. Monolayers infected with *Salmonella sopB*^3xFLAG^ were fixed and immunostained for bacteria (αCSA) and effector (αFLAG). (A) The percentage of effector-positive infected cells was quantified. Means ± SD from 3 independent experiments. (B) Representative confocal images of *Salmonella*-infected HeLa cells with SopB^3xFLAG^ staining. Scale bars: 10 μm; inset 2 μm. (C) Analysis of intracellular *Salmonella* populations in SopB^3xFLAG^-positive cells. Effector-positive cells were classified according to the presence or absence of cytosolic bacteria. Means from 3 independent experiments. Statistical significance was analyzed by unpaired Student’s *t*-test. *** P ≤ 0.001, ns = not-significant. (D) Representative confocal images of HeLa cells infected with *Salmonella sopB*^3xFLAG^ subjected to differential permeabilization. Cytosolic (Cyto.) bacteria (red) and the cytosolic tail of calnexin (yellow, permeabilization control) were labeled after digitonin permeabilization. Total bacteria (grey) and SopB^3xFLAG^ (green) were detected post saponin permeabilization. Scale bars: 10 μm; inset 2 μm. See also S1 Fig.

Since SopB-dependent Akt phosphorylation was detected in cells containing >20 bacteria at 6 hpi we next wanted to: confirm the presence of SopB in this subpopulation; determine its subcellular localization; and show whether it correlates with the presence of cytosolic or vacuolar bacteria. Detection of T3SS effectors, such as SopB, in host cells is difficult due to the low amounts delivered as well as a lack of good antibodies. For these reasons, we used epitope tagged SopB for these experiments. Specifically, we used WT *Salmonella* expressing 3xFLAG-tagged SopB (SopB^3xFLAG^) from the *sopB* chromosomal locus and under the control of its native promoter (*Salmonella sopB*^3xFLAG^). Infected HeLa cells were fixed and stained with an anti-FLAG antibody. Under the conditions used here (paraformaldehyde fixation and permeabilization with saponin) antibodies cannot cross the bacterial cell wall so that intrabacterial SopB^3xFLAG^ is not detected. At 1 hpi, ~20–30% of infected cells contained detectable levels of extrabacterial SopB^3xFLAG^ (SopB^+^ cells) (Fig 2A and 2B). Thereafter, the number of SopB^+^ cells rapidly decreased so that by 2 hpi, less than 10% of infected cells were SopB^+^ and this did not change significantly up to 8 hpi. At 6 hpi, similar to pAkt, SopB^3xFLAG^ was predominantly detected in cells containing >20 bacteria. In cells infected with the Δ*sopB* strain, no significant FLAG staining was observed at any time point even in cells containing hyper-replicating bacteria. Interestingly, SopB^3xFLAG^ staining was predominantly detected in close proximity to individual bacteria whereas pAkt was distributed throughout the cytosol (compare Figs 1C and 2B).

In our experience, cytosolic bacteria are readily distinguished from vacuolar bacteria at later time points (4–8 hpi) due to their greater intracellular numbers. However, individual cells are often infected with more than one bacteria and can harbor both vacuolar and cytosolic bacteria. Therefore, in order to definitively determine whether SopB^3xFLAG^ staining is associated with cytosolic or vacuolar bacteria, and how this changes with time, we used digitonin-based differential permeabilization (a.k.a. phagosomal protection assay) [8]. Selective permeabilization of the plasma membrane, while leaving the phagosome membrane intact, allows cytosolic bacteria to be selectively stained with antibodies. Cells were infected with *Salmonella sopB*^3xFLAG^, or Δ*sopB* strain as a control. Cells containing one or more bacteria and staining positive for SopB^3xFLAG^ were categorized into two populations: 1) Infected cells containing only vacuolar bacteria, and 2) Infected cells containing any cytosolic bacteria. At 1 hpi, the T3SS1 effector was found in both populations (Fig 2C and 2D). In contrast, at 6 hpi, SopB^3xFLAG^ staining was almost exclusively detected in cells containing cytosolic hyper-replicating bacteria. To determine whether this is specific to SopB we performed similar experiments with another T3SS1 effector, SipA, and obtained almost identical results (S1 Fig). Thus, at 6 hpi, the presence of T3SS1 effectors in infected cells strongly correlates with the presence of hyper-replicating cytosolic *Salmonella* reinforcing that it is this population, rather than vacuolar bacteria, which delivers the effectors into the host cell.

### Association of T3SS1 effectors with cytosolic *Salmonella* in epithelial cells *in vivo*

**Fig 3:**
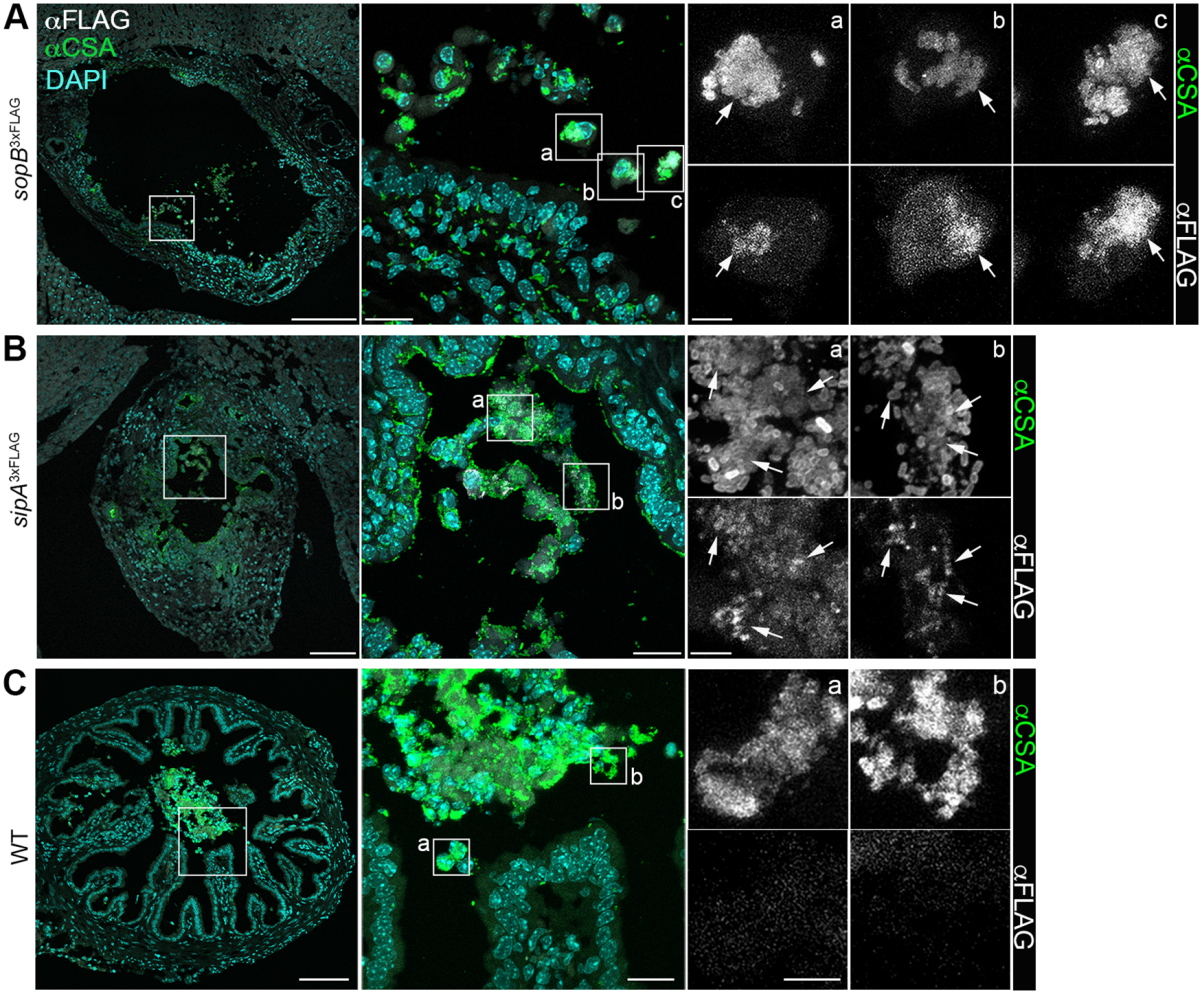
T3SS1 effectors are detected in murine gallbladder epithelial cells harboring hyper-replicating *Salmonella*. Representative confocal images of gallbladders from mice infected with *Salmonella* expressing SopB^3xFLAG^ (A), SipA^3xFLAG^ (B), or no FLAG-tagged effector (C). Cryosections were immunostained for effector (αFLAG, grey) and bacteria (αCSA, green). DNA was stained with DAPI (cyan). Arrows indicate effector labeling. Whole gallbladder image (Scale bar: 200 μm) was compiled from tiled images; boxed areas are shown as enlarged overlay (scale bar: 20 μm) and single channel images (Scale bar: 5 μm). See also S2 Fig.

The above experiments revealed that, in HeLa cells, T3SS1 effectors are present in the subpopulation of cells that contain cytosolic hyper-replicating *Salmonella*. To investigate whether this phenotype occurs *in vivo*, we utilized a C57BL/6J mouse model in which *Salmonella* disseminates to various tissues, including the gallbladder. Cytosolic hyper-replication has been observed in gallbladder epithelial cells which are extruded into the lumen where they can be readily identified [8]. In order to detect the T3SS1 effectors, mice were infected with *Salmonella* expressing either SopB^3xFLAG^ or SipA^3xFLAG^ from their chromosomal loci. Gallbladders harvested 5 days pi were stained for *Salmonella* and FLAG-tagged effectors. Extruded epithelial cells containing cytosolic, hyper-replicating *Salmonella* were observed in the lumen and many of these cells stained for SopB^3xFLAG^ or SipA^3xFLAG^ (Fig 3A, 3B and S2 Fig). In contrast, there was no significant FLAG staining in cells containing hyper-replicating WT *Salmonella* (no FLAG-tagged effectors, Fig 3C). Thus, *in vivo*, SopB and SipA are present in extruded epithelial cells containing cytosolic *Salmonella*.

### *De novo* synthesis and delivery of SopB in cytosolic bacteria is required for Akt phosphorylation

**Fig 4:**
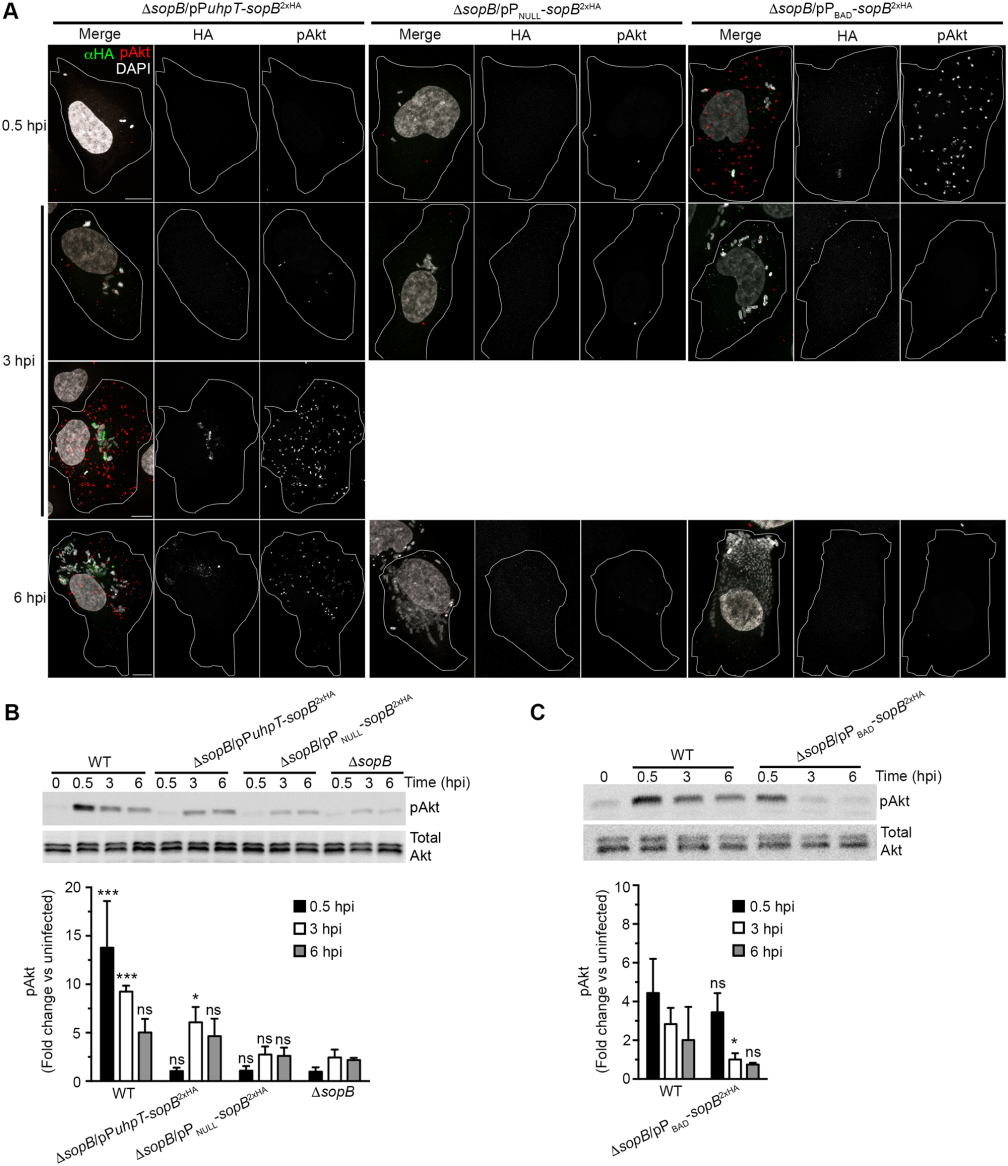
Expression of SopB in cytosolic bacteria is sufficient to induce post-invasion Akt phosphorylation. HeLa cells were infected with Δ*sopB Salmonella* complemented with plasmid borne *sopB* under the control of a promoter that is induced in the presence of glucose-6-phospate (P_*uhpT*_), or the arabinose inducible reporter (P_BAD_). (A) Induction of SopB expression in cytosolic bacteria stimulates Akt phosphorylation. Cells were fixed, and processed for PLA of phospho-Akt (pAkt) and immunostaining of SopB-^2xHA^ (αHA). DNA was stained with DAPI. Representative confocal images are shown. Scale bars: 10 μm. (B, C) Western blot for total Akt and phosphorylated Akt (pAkt), and band densitometry, normalized to uninfected, for phosphorylated Akt, Densitometry data show means ± SD for 3 independent experiments. Western blot is from one representative experiment. Statistical significance was analyzed by 2-way ANOVA with Bonferroni’s post-test and compared to corresponding time points of Δ*sopB* (B) or WT (C) infected cells. * P ≤ 0.05, *** P ≤ 0.001, ns = not-significant.

To separate the post-invasion role of SopB in the cytosolic population of *Salmonella* from its role in invasion we used an inducible gene expression system in the Δ*sopB* background. The hexose phosphate transporter promoter, P*uhpT*, responds to exogenous glucose-6-phosphate, a glucose metabolite that is exclusively found in the cytosol [21,22]. To first verify the fidelity of P*uhpT* in our system, we used a plasmid borne GFP transcriptional reporter, pP*uhpT-gfp*, in WT *Salmonella*. These bacteria were internalized into HeLa cells and then the differential permeabilization assay and confocal microscopy used to determine their intracellular localization and GFP status (S3 Fig). At all time points GFP^+^ bacteria were found exclusively in the cytosol confirming that P*uhpT* is specifically induced in this subpopulation of intracellular *Salmonella*. Next, we used a transcriptional fusion of this cytosol inducible promoter with HA-tagged *sopB*, P*uhpT-sopB*^2XHA^, in a Δ*sopB* background to assess the role of SopB in cytosolic bacteria. As expected, immunostaining for SopB^2xHA^ in infected cells showed an expression pattern similar to GFP under P*uhpT*, confirming that the effector was expressed only in cytosolic *Salmonella* (Fig 4A). Using PLA to detect pAkt revealed a dramatic increase in pAkt in SopB^2xHA^-positive cells, particularly in cells with cytosolic hyper-replicating bacteria at 6 hpi. In contrast, infected cells lacking bacterial SopB^2xHA^ expression were devoid of pAkt. To confirm that there was no SopB activity at early time points in this system, lysates prepared from cells infected with Δ*sopB*/pP*uhpT-sopB*^2xHA^ were assessed by Western blotting (Fig 4B). At 0.5 hpi, the pAkt levels were similar to those infected with the Δ*sopB* strain and uninfected cells whereas at 3 and 6 hpi, pAkt levels were 2.5- and 2-fold higher than Δ*sopB* infected cells, respectively, and were comparable to WT infected cells. Increased Akt phosphorylation was SopB-dependent since there was no increase in cells infected with Δ*sopB* bearing a promoterless control plasmid (pP_NULL_-*sopB*^2xHA^) (Fig 4A and 4B). These results illustrate that post-invasion cytosolic induction of SopB expression is sufficient for late Akt phosphorylation in infected cells.

The above experiments showed that, in the absence of SopB during invasion, *de novo* production of SopB by cytosolic bacteria could account for SopB-dependent Akt phosphorylation at 6 hpi. However, we also wanted to know whether, in the absence of cytosolic SopB, persistence following invasion could also possibly contribute to Akt phosphorylation. For this, we used a strain (Δ*sopB*/pP_BAD_-*sopB*^2xHA^) with a plasmid borne arabinose inducible construct. Expression of the tagged effector (SopB^2xHA^) was induced immediately prior to invasion by addition of arabinose during growth of *Salmonella* under SPI1-inducing conditions. Following internalization in HeLa cells, SopB^2xHA^ should be rapidly depleted due to the absence of arabinose in the intracellular environment. Immunofluorescence microscopy and Western blot analysis of HeLa cells infected with Δ*sopB*/pP_BAD_-*sopB*^2xHA^ showed SopB^2xHA^ expression and elevated pAkt only at 0.5 hpi, but not at 3 and 6 hpi (Fig 4A and 4C). Thus, SopB delivered during invasion does not persist and, therefore, does not sustain Akt phosphorylation. Altogether, these results indicate that SopB delivered by cytosolic bacteria post-invasion is both essential and sufficient for sustained Akt phosphorylation in infected HeLa cells.

### SopB prolongs the lifespan of HeLa cells containing cytosolic hyper-replicating *Salmonella*

**Fig 5:**
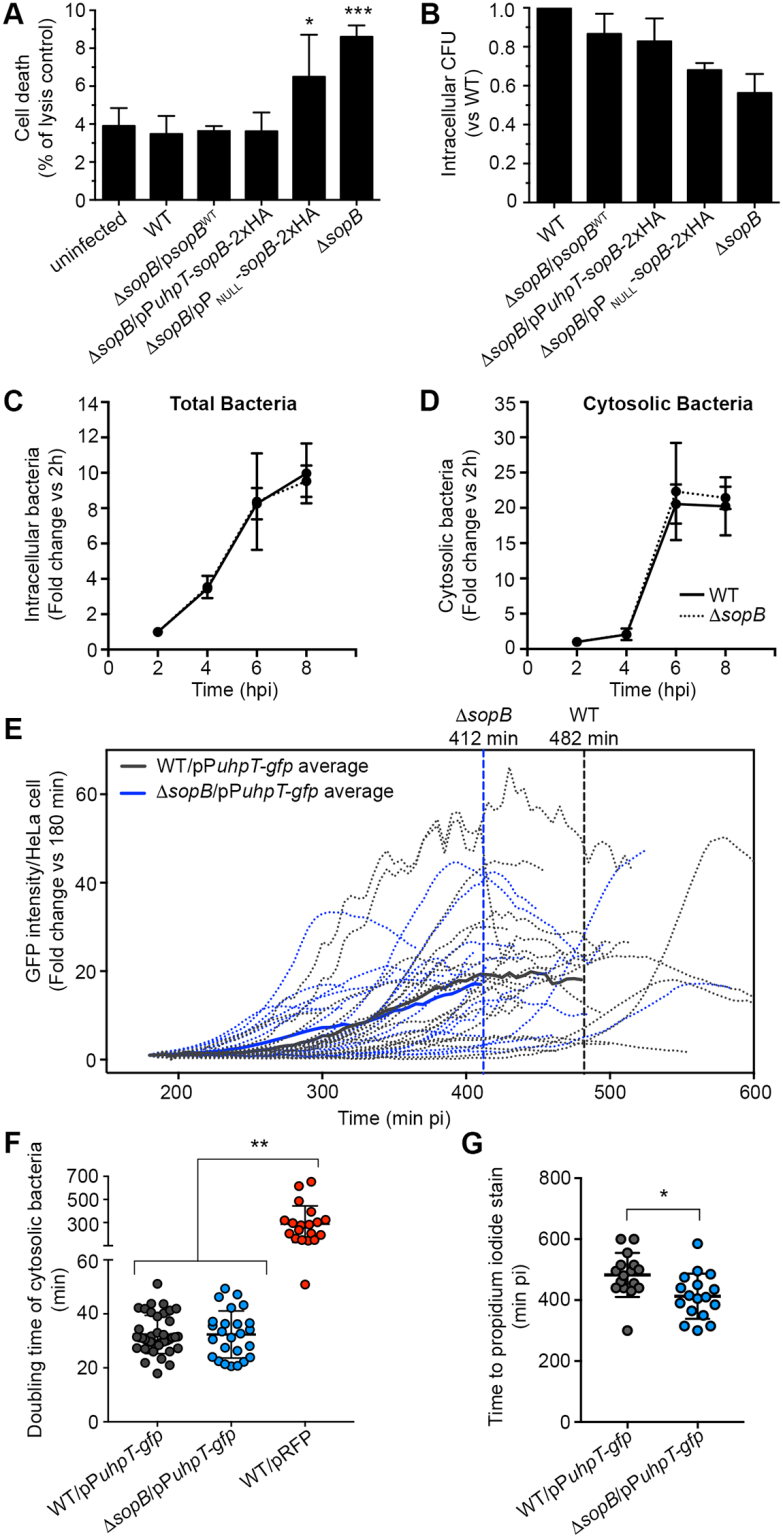
SopB promotes survival of cells containing cytosolic *Salmonella*. (A, B) Cell death (A, LDH assay) and intracellular bacterial loads (B, gentamicin protection assay) at 6 hpi in HeLa cells infected with *Salmonella* WT, the Δ*sopB* strain, or plasmid complemented Δ*sopB* strain. Means ± SD of 3 independent experiments. Statistical significance was analyzed in comparison to WT infected cells. (C, D) Comparison of total and cytosolic *Salmonella* WT and Δ*sopB* in HeLa cells determined by gentamicin protection assay from untreated (total bacteria) or choloroquine treated cells (cytosolic bacteria). Means ± SD of 3 independent experiments. (E-G) Quantification of cytosolic replication and host cell death by live cell imaging of HeLa cells containing cytosolic WT and Δ*sopB* strains. To positively identify cytosolic bacteria the P*uhpT*-*gfp* reporter was used. The total green fluorescent signal per HeLa cell was measured. To detect cell death, propidium iodide was added to the culture media at the beginning of imaging. The mean time to PI staining of nuclei is indicated for cells containing cytosolic WT and Δ*sopB* strains. Statistical significance in (F, G) was analyzed by 1-way ANOVA with Bonferroni’s post-test and unpaired Student’s *t*-test, respectively. * P ≤ 0.05, ** P ≤ 0.01, *** P ≤ 0.001.

We have previously found that SopB could protect infected HeLa cells from apoptosis [15], although at that time we assumed that intracellular replication of *Salmonella* occurred within the SCV. The data presented above suggests that the anti-apoptotic effect of SopB could be limited to cells containing cytosolic hyper-replicating *Salmonella*. To address this question, we measured release of the cytosolic enzyme lactate dehydrogenase (LDH) from monolayers of HeLa cells infected with WT or Δ*sopB Salmonella* at 6 hpi (Fig 5A and 5B). At this time point, cells infected with the WT strain had similar levels of lysis (3.5±0.9%) as uninfected cells whereas the cells infected with the Δ*sopB* strain showed a two-fold increase in cell lysis (8.6±0.6%), despite lower numbers of intracellular of Δ*sopB* relative to WT (Fig 5A and 5B). In contrast, no increase in lysis was observed in cells infected with the Δ*sopB* strain complemented with plasmid borne SopB, either under the control of its own promoter (p*sopB*^WT^) or the uhpT promoter (pP*uhpT*-*sopB*-^2xHA^). A control “promoter-less” plasmid (pP_NULL_-^sopB-2xHA^) did not rescue this phenotype. Considering that only 10–15% of infected cells contain hyper-replicating cytosolic *Salmonella* (Fig 1 [8]) and that not all HeLa cells in the monolayer are infected, the relatively small total amount of lysis caused by strains lacking SopB is not unreasonable. The lower numbers of Δ*sopB* bacteria observed at 6 hpi (Fig 5B) as well as other studies suggested that SopB may contribute to intracellular replication of *Salmonella* [17]. However, we were unable to detect any defect in intracellular replication of the Δ*sopB* strain when the results were normalized to the number of intracellular bacteria at 2 hpi (Fig 5C and 5D). In order to gain a better understanding of the role of SopB in cells containing hyper-replicating cytosolic bacteria we focused our analysis on this specific sub-population of cells by using a live cell imaging approach previously used to compare the replication rates of cytosolic and vacuolar *Salmonella* [9]. In order to assess cytosolic replication we took advantage of the plasmid borne GFP transcriptional reporter, pP*uhpT-gfp*, described above (Fig 4). Infected HeLa cells were imaged on a spinning disc confocal system for up to 10 h pi (Fig 5E, 5F and 5G). As a rapid readout for cell death, propidium iodide was used. This red fluorescent nuclear and chromosome counterstain is not permeant to live cells and is commonly used to detect dead cells. Post acquisition analysis of time lapse movies showed that cells containing the Δ*sopB* strain died earlier (412 min pi) than those containing WT *Salmonella* (482 min pi) (Fig 5E and 5G). In contrast, there was no detectable difference in the doubling rates of the two strains (Fig 5F). As a control for doubling rate, we included a strain in which constitutive expression of red fluorescent protein (RFP) causes a defect in intracellular replication. Altogether these results show that SopB does not affect intracellular replication of *Salmonella* but rather promotes the survival of HeLa cells containing hyper-replicating cytosolic *Salmonella*.

### SPI1 is widely induced in cytosolic hyper-replicating *Salmonella*

**Fig 6:**
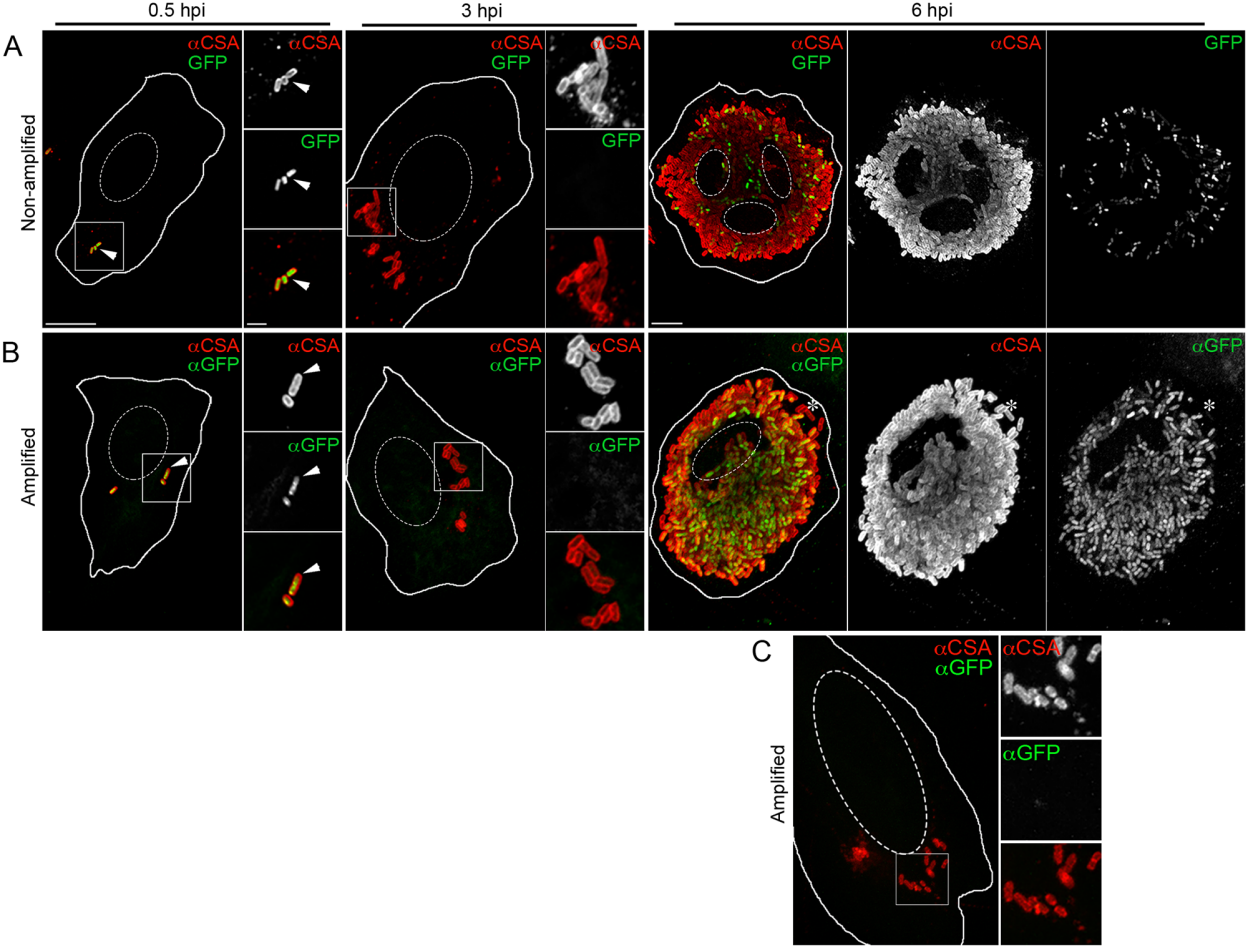
Cytosolic replication of *Salmonella* correlates with widespread SPI1 expression. HeLa cells were infected with WT *Salmonella* bearing a plasmid borne transcriptional reporter for the SPI1 gene *prgH* (P*prgH*-gfp[LVA], A-C). Cells were fixed with PFA (A, Non-amplified), or methanol (B and C, Amplified), and immunostained for bacteria (αCSA, red); methanol fixed cells were also stained for GFP (αGFP, green). Representative confocal images of cells containing cytosolic hyper-replicating (A, B) or vacuolar (C, <20 bacteria/cell) bacteria are shown. Magnified insets show single-channel images of boxed area. Nuclei and cell boundaries are outlined. Scale bars: 10 μm; inset 2 μm.

Previous studies have shown that some, but not all, cytosolic hyper-replicating bacteria are SPI1 induced [8,12]. Since this would have significant implications for our findings that SopB-dependent Akt phosphorylation is widespread in this population of cells, we considered whether this was an accurate estimation of SPI1 induction. One possibility is that limitations in the sensitivity of the experimental systems could result in an underestimation of SPI1 induction. Specifically, HeLa cells were infected with *Salmonella* containing a GFP-*prgH* transcriptional reporter with destabilized GFP, GFP[LVA] (P*prgH*-gfp[LVA]) [8,12]. As shown in Fig 6A, this system reveals many SPI1-induced (GFP^+^) bacteria at 0.5 hpi and, thanks to the short half-life of the destabilized GFP[LVA], down-regulation of SPI1 results in a dramatic decrease in the number of GFP^+^ bacteria by 3 hpi. By 6 hpi, GFP fluorescence reappears, although only a fraction of hyper-replicating cytosolic bacteria are GFP^+^, suggesting that SPI1 induction is not universal in this population ([8] and Fig 6A). Nevertheless, we considered that, while the use of destabilized GFP[LVA] is critical for following down-regulation of the *prgH* promoter, it could also result in the under-detection of SPI1-induced bacteria. Therefore, to increase the sensitivity of detection without losing temporal fidelity, we used an anti-GFP antibody to amplify the fluorescent signal (Fig 6B). One caveat of this system is that, in order to get the antibodies into the bacteria, we had to permeabilize with methanol fixation, which resulted in denaturation of GFP and, consequently, loss of fluorescence. However, antibody recognition was not affected and thus the antibody-mediated amplification revealed a dramatic increase in the proportion of cytosolic hyper-replicating bacteria that were GFP^+^ at 6 hpi (Fig 6, compare A and B). The fidelity of the amplified system was confirmed by the lack of any detectable increase in GFP staining in intracellular populations of bacteria that are not SPI1 induced (see 3 h time point and vacuolar bacteria at 6 h, Fig 5B and 5C). Thus, by 6 hpi, T3SS1 is widely expressed in cytosolic hyper-replicating, but not vacuolar, *Salmonella*.

### T3SS1-dependent delivery of SopB and SipA by cytosolic *Salmonella*

**Fig 7:**
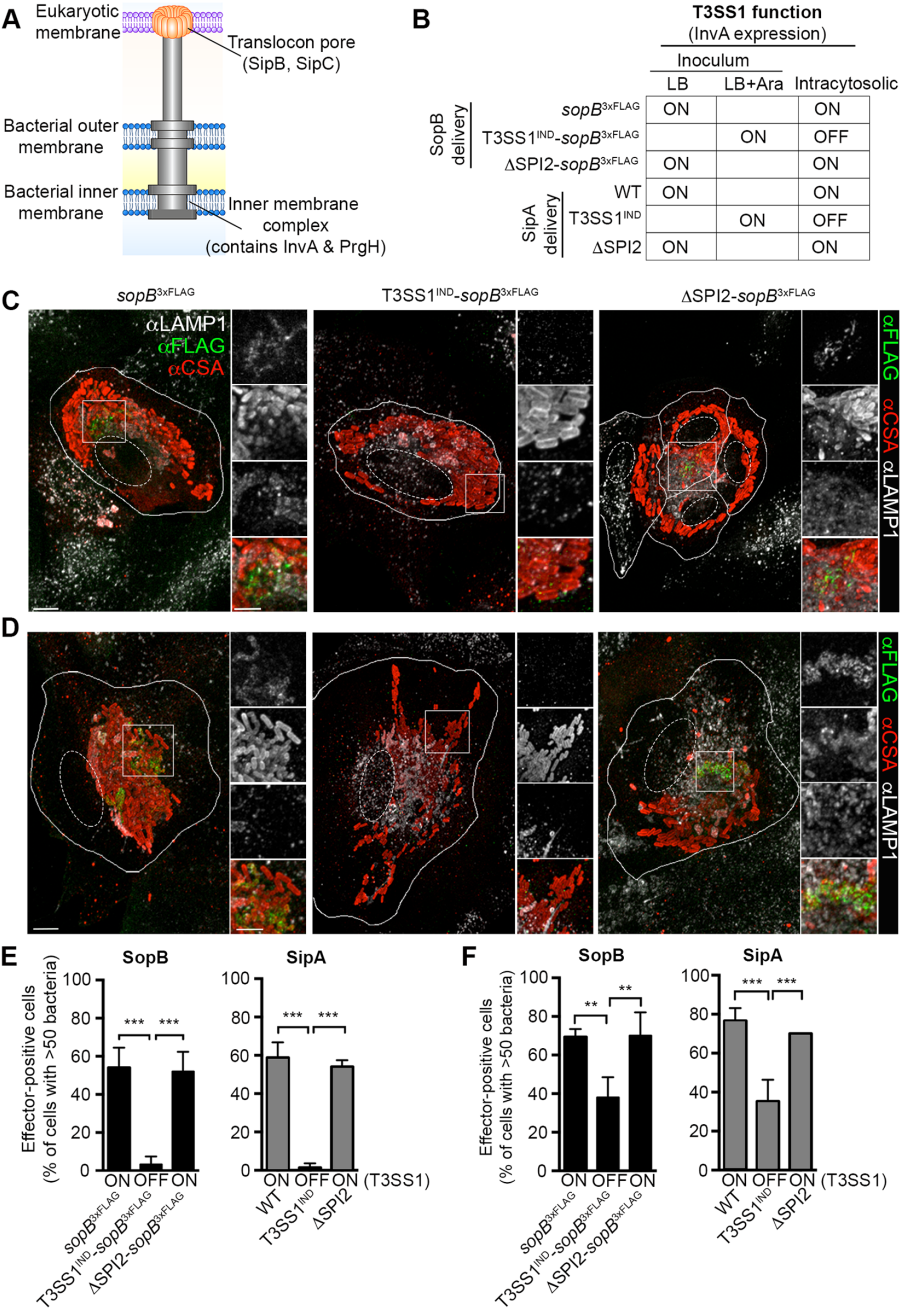
T3SS1 is required for delivery of SopB and SipA by cytosolic *Salmonella*. (A) Model of T3SS1. (B) Key to *Salmonella* strains used to determine requirement for T3SS1 and T3SS2 in effector delivery by cytosolic bacteria. Representative confocal images of infected HeLa (C) and C2BBe1 (D) cells stained for SopB^3xFLAG^. Infected monolayers were PFA fixed at 6 hpi and stained for effector (αFLAG), bacteria (αCSA) and LAMP1 (αLAMP1). Scale bars: 10 μm; inset 2 μm. The percentage of HeLa (E) or C2BBe1 (F) cells, containing >50 bacteria, with SopB or SipA staining. Means ± SD of 3 independent experiments. ** P ≤ 0.01, *** P ≤ 0.001. See also S4A–S4E Fig.

During invasion of epithelial cells, SPI1 effectors, including SopB and SipA, are translocated across the plasma membrane by the T3SS1. In the absence of a functional T3SS1, bacterial internalization into HeLa cells is almost completely abrogated, making it technically challenging to study the role of T3SS1 in post invasion events [23]. To circumvent this problem, we developed an inducible strain, which has a functional T3SS1 for invasion, but is essentially a T3SS1 defective mutant in the intracellular environment. To do this we used the arabinose-inducible promotor *P*_*BAD*_ to control expression of the SPI1 gene *invA*, which encodes a conserved structural component of T3SS1 (Fig 7A) [24]. This T3SS1 arabinose-inducible strain, T3SS1^IND+^-*sopB*^3xFLAG^, was invasion competent when grown under SPI1-inducing conditions in the presence of arabinose (S4A Fig) but was unable to deliver detectable amounts of either of the T3SS1 effectors (SopB^3xFLAG^, SipA) in cells containing cytosolic hyper-replicating *Salmonella* (>50 bacteria/cell) (Fig 7). In these, and subsequent, experiments we used a higher stringency cut-off for cytosolic bacteria (>50 bacteria/cell) so as to avoid any possibility of contamination by cells containing only vacuolar bacteria. To confirm that the defect in effector delivery by T3SS1^IND^ was not due to a lack of effector expression, we immunostained intracellular bacteria following methanol-permeabilization. Levels of intrabacterial effector staining for the T3SS1^IND^ and WT strains were comparable (S4B–S4E Fig). We also tested whether the SPI2 encoded T3SS2 could be involved in SopB or SipA delivery since some T3SS1 effectors, including SopB, have the potential to be delivered by T3SS2 as well as T3SS1 [25,26]. However, a T3SS2 deficient strain (ΔT3SS2) delivered SopB^3xFLAG^ and SipA at levels indistinguishable from *Salmonella* WT (Fig 7C and 7E). Similar results, showing a requirement for T3SS1 but not T3SS2, were obtained in C2BBe1 cells (Fig 7D and 7F). Thus, T3SS1, but not T3SS2, is required for delivery of the effector proteins SopB and SipA by cytosolic hyper-replicating *Salmonella*.

### The T3SS1 translocon is not required for delivery of SopB and SipA by cytosolic *Salmonella*

**Fig 8:**
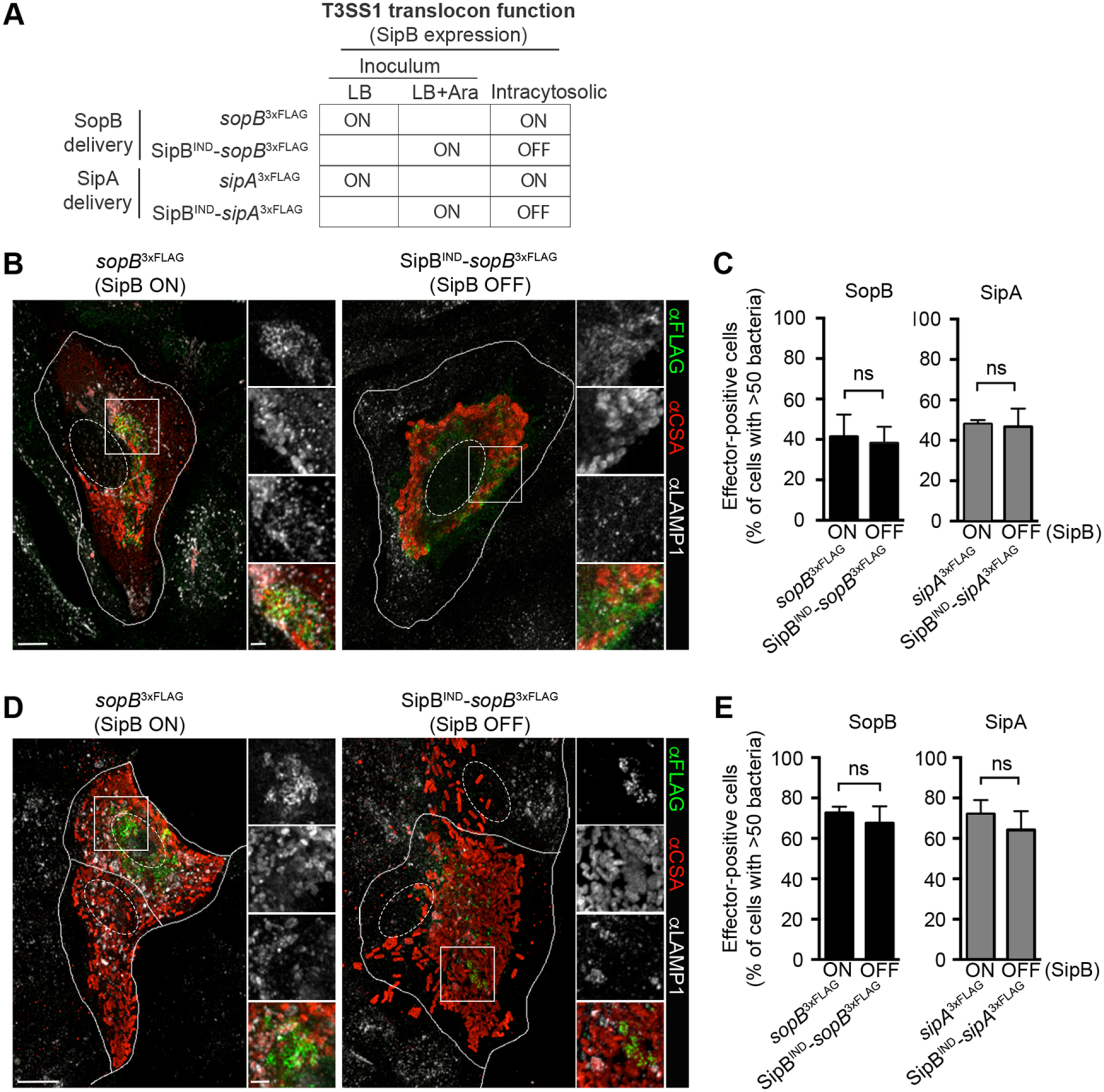
SipB is dispensable for T3SS1 effector delivery by cytosolic *Salmonella*. (A) Key to *Salmonella* strains used to determine requirement for the T3SS1 translocon in effector delivery by cytosolic bacteria. (B-E) Representative confocal images of infected HeLa (B) and C2BBe1 (D) cells, with quantification of effector delivery shown in (C) and (E), respectively. The percentage of infected cells containing >50 bacteria with effector staining was scored. Monolayers were infected, fixed at 6 hpi and immunostained as described in Fig 7. Means ± SD of 3 independent experiments. ns = not-significant. Scale bars: 10 μm; inset 2 μm. See also S4F–S4I Fig.

Canonical delivery of effectors into host cells via T3SSs requires contact with host membrane and subsequent formation of a translocon pore through which the effectors are delivered. For T3SS1, the translocator proteins, SipB and SipC, are required to establish the translocon pore and translocation does not occur in their absence (see illustration in Fig 7A) [27–29]. Immunostaining for SipB in WT *Salmonella* infected cells revealed this translocon component in association with cytosolic but not vacuolar bacteria at 6 hpi, corroborating the induction of T3SS1 in cytosolic *Salmonella* (S5 Fig). To examine whether delivery of SopB and/or SipA by cytosolic *Salmonella* requires the T3SS1 translocon, we took advantage of a translocation defective mutant (Δ*sipB*), which is unable to invade epithelial cells (S4F Fig) but retains the ability to secrete effectors in broth culture (S4G and S4H Fig). To generate an invasion-competent *sipB* mutant strain, we again used the *P*_*BAD*_ inducible system so that *sipB* expression was induced when bacteria were grown under SPI1-inducing conditions in the presence of arabinose (SipB^IND^) (S4I Fig). Confocal microscopy was used to assess the ability of the SipB^IND^ strain to deliver SopB^3xFLAG^ and/or SipA^3xFLAG^ in epithelial cells. At 6 hpi, effector staining in infected HeLa cells infected with WT or SipB^IND^ bacteria revealed no difference in the numbers of cells containing cytosolic hyper-replicating *Salmonella* (>50 bacteria/cell) that were positive for effectors (~40–50%, Fig 8B and 8C). Immunostaining for SipB in SipB^IND^ infected HeLa cells confirm expression at 0.5 hpi but not at 3 and 6 hpi, excluding the contribution of residual SipB following invasion (S5 Fig). Similar results were obtained in C2BBe1 cells (Fig 8D and 8E). Thus, the T3SS1 translocon pore is not required for SopB or SipA delivery by cytosolic bacteria.

## Discussion

**Fig 9:**
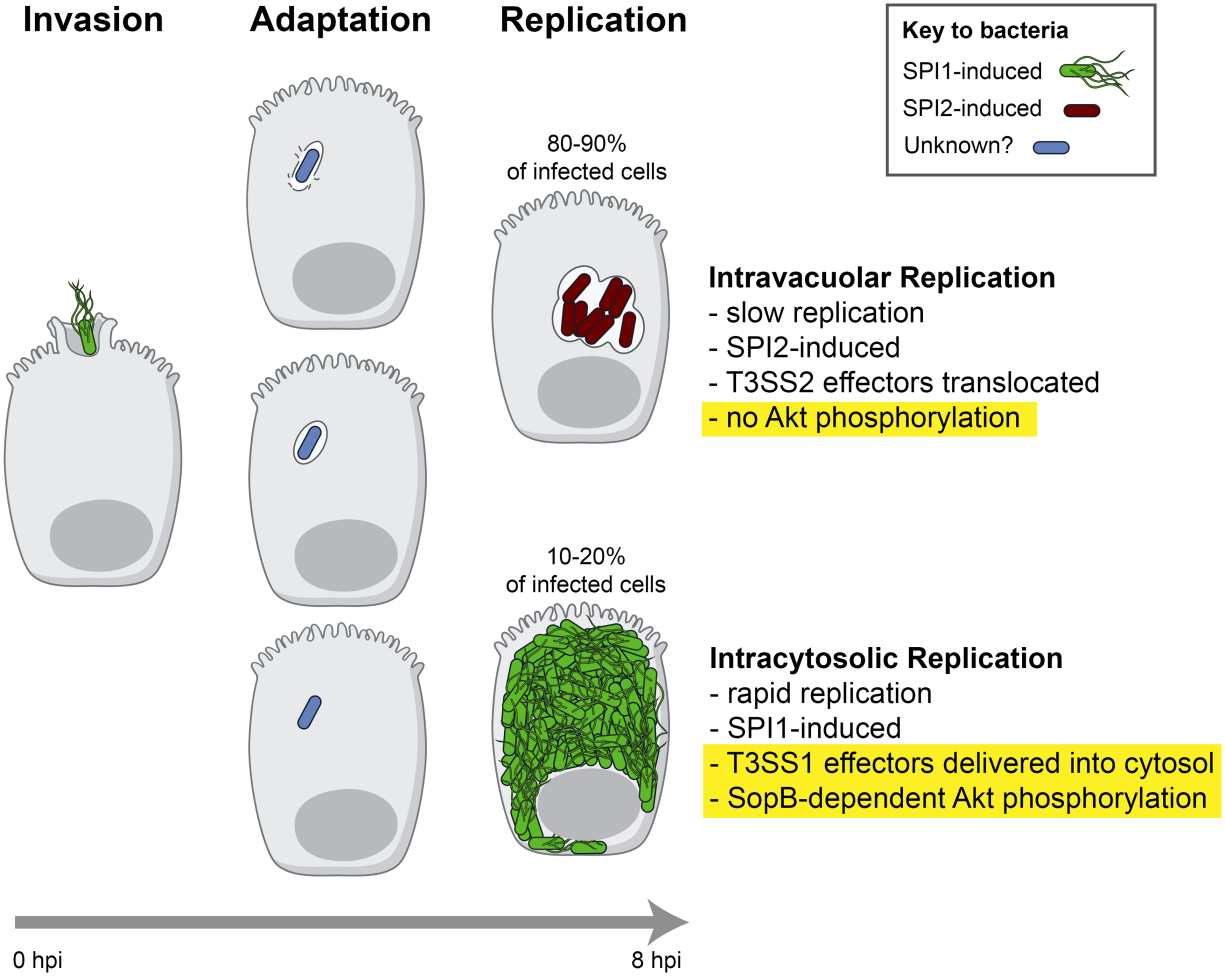
Schematic of the distinct subpopulations of intracellular *Salmonella* during infection of epithelial cells. SPI1-induced *Salmonella* invade non-phagocytic cells by translocating T3SS1 effectors into host cells. The majority of internalized bacteria remain within SCVs, although some escape into the cytosol. The mechanism of escape is not well understood but may be the result of damage by T3SS1 needles during invasion [30]. Subsequent adaptation to these two microenvironments (vacuolar vs cytosol) ultimately leads to physiologically and transcriptionally distinct intracellular bacterial populations. In particular, hyper-replicating cytosolic bacteria are SPI1 induced and secrete at least two T3SS1 effectors (SopB and SipA) into the cytosol where they can potentially target host cell pathways. Here we demonstrated that SopB-dependent Akt phosphorylation extends the lifespan of HeLa cells containing cytosolic bacteria. Findings from this study are highlighted in yellow.

The ability of *Salmonella* to actively invade and colonize epithelial cells is critical for pathogenesis. Following invasion, the bacteria can survive and replicate within the SCV, a modified phagosome, or in the cytosol. Effectors translocated by the SPI1-encoded T3SS1 play critical roles in invasion. Here, we show a novel role for T3SS1 in the cytosolic subpopulation of intracellular bacteria (See model in Fig 9). Delivery of the effector SopB, via a translocon-independent mechanism, leads to Akt phosphorylation and prolonged survival of epithelial cells containing cytosolic *Salmonella*.

In enteric infection, environmental signals in the lumen of the small intestine—though still not well understood—are believed to be essential for SPI1 induction [31,32]. Our findings show that induction can also occur in the intracellular environment, specifically the cytosol of infected epithelial cells. This must confer an advantage to the bacteria since expression of the SPI1-encoded T3SS1, and its associated effectors, comes at a cost [33]. The T3SS1 needle/apparatus is recognized in the cytosol of mammalian cells resulting in inflammasome activation and host cell death [34]. In epithelial cells, the SPI1-encoded T3SS1 is specifically expressed in the cytosolic subpopulation of bacteria [8], leading to cell death via activation of a non-canonical inflammasome [11,35]. Whether this is an advantage to the host or pathogen may depend on the context. Is SPI1-induction in these bacteria priming them for the extracellular environment, i.e. lumen of small intestine, or is there a specific function for T3SS1 in the cytosolic niche? Here, we show that the latter may be true since delivery of T3SS effectors delays the onset of cell death thus conferring an advantage to the intracellular pathogen.

The activity of Akt, a central regulator of eukaryotic cell death and survival, is regulated by phosphorylation. *Salmonella* uses SopB to target this pro-survival kinase suggesting an important role for T3SS1 in regulating host cell survival [14,15,36]. However, since SopB is not essential for intracellular replication, the role of SopB-dependent Akt phosphorylation in epithelial cells was still unclear. In order to resolve this disparity, we used a single-cell approach to re-examine the temporal relationship between SopB and Akt phosphorylation. We found that, at later time points, Akt phosphorylation was highly induced in the subpopulation of infected cells containing hyper-replicating cytosolic *Salmonella*. *De novo* T3SS1-dependent delivery of SopB by cytosolic bacteria, as opposed to persistence of the effector, was key to this second wave of Akt phosphorylation. As a master regulator, Akt affects diverse cellular processes and lies at the crossroads between cell survival and death [37]. Dysregulation has been implicated in many human cancers and recently, an interesting link between *Salmonella*, Akt and human cancer was described [38]. The incidence of gallbladder cancers is increased in areas where there is high incidence of *Salmonella* Typhi infection, and experiments in chronically infected mice showed that tumors are induced in a SopB-dependent manner, suggesting a causal relationship [39]. This is consistent with our finding showing that SopB-dependent Akt activitation does not directly affect the ability of *Salmonella* to replicate but rather delays host cell death [15,40–43]. This may be a more widespread strategy since the PI3K-Akt signaling cascade is also targeted by other bacterial pathogens, including *Chlamydia trachomatis*, *Chlamydia pneumonia*, *Coxiella burnettii* and *Mycobacterium tuberculosis*, to delay or prevent apoptosis in infected cells [44–47].

Previously, the continued presence of T3SS1 effectors post-invasion has been explained by effector persistence in the intracellular environment [48] or delivery by the T3SS2 [25,26,49]. The main reason that the role of T3SS1 post-invasion is not well understood is because mutants lacking T3SS1 activity are unable to infect host cells. To overcome this obstacle we developed a set of inducible mutants, which allowed us to specifically address the post-invasion roles of the T3SS1 and SopB. Using this approach yielded a more complete picture of the differences between the two intracellular populations of *Salmonella* and particularly the activity of T3SS1 in the cytosolic population. SopB delivery by cytosolic bacteria requires continued transcription and translation of the molecule by the bacterium post-invasion [50]. Furthermore, T3SS2 has no role in delivery of SopB or SipA to infected host cells, although that has previously been suggested as a mechanism for sustained delivery both *in vitro* and *in vivo* [25,26,49]. Using a murine systemic infection model, we confirmed that SPI1 effectors are present in cells containing large numbers of cytosolic bacteria *in vivo*. Altogether, our data show that T3SS1 can account for the delivery of T3SS1 effectors at later stages of infection.

To the best of our knowledge this is the first time that T3SS1 has been shown to use a translocon-independent mechanism of delivery. Cytosolic *Salmonella* are not contained within a membrane-bound compartment, have no obvious contact with a membrane and can deliver effectors in the absence of the translocator protein SipB. For both *Salmonella* T3SS1 and the *Shigella flexneri* Mxi-Spa T3SS, the translocon proteins are gatekeepers that effectively prevent secretion of effectors in the absence of a specific signal. Mutants lacking translocon proteins constitutively secrete effectors but are unable to invade host cells [51,52]. Interestingly, *Shigella*, which also replicates in the cytosol of epithelial cells, can uncouple translocation and secretion depending on environmental stimuli [53]. Whether the same is true for *Salmonella* is still to be determined. The *Salmonella* SPI1, *Shigella* Mxi-Spa and *Yersinia* Ysa T3SSs are members of the Inv/Mxi-Spa subfamily, and other members are found in a number of pathogens including, *Burkholderia pseudomallei* (Bsa) and *Chromobacterium violaceum* (Cpi-1/-1a) [1,54]. While these T3SSs generally have key roles in the invasion of non-phagocytic cells [55–58], several of them also have post-invasion activities that contribute to pathogenesis [34,59,60].

SPI1 is a paradigm for bacterial adaptation to the host environment [61]. Here, we identified a novel activity of the SPI1-encoded T3SS1 in the cytosol of mammalian epithelial cells. Thus, this highly regulated T3SS mediates host cell interactions in at least two vastly different environments: the lumen of the gut, and the cytosol of epithelial cells. Given the central role of T3SSs at the host-pathogen interface, our findings highlight the need to reassess the contributions of the *Salmonella* T3SS1 and its effectors in post-invasion events to fully understand their impact on bacterial pathogenesis.

## Materials and methods

### Bacterial strains and mammalian tissue culture cell lines

Bacterial strains, plasmids and oligonucleotides used in this study can be found in the supporting tables document. All mutants are derivatives of the parental *Salmonella* Typhimurium strain SL1344 [62] and were constructed using the bacteriophage λ recombinase system [63]. Bacteria were cultured in Miller formulation lysogeny broth (LB-M) supplemented with appropriate antibiotic, where necessary.

HeLa cells (human cervical adenocarcinoma, ATCC CCL-2) and the Caco-2 subclone C2BBe1 (human colorectal adenocarcinoma, ATCC CRL-2102) were grown at 37°C in 5% CO_2_ in complete growth medium (CGM): HeLa—MEM supplemented with 10% (v/v) heat-inactivated fetal bovine serum (Invitrogen), 2 mM L-Glutamine and 1 mM sodium pyruvate; C2BBe1—DMEM supplemented with 10% (v/v) heat-inactivated fetal bovine serum, 4 mM L-Glutamine and human transferrin (10 μg/mL). Both cell lines were passaged as recommended by ATCC and used within 15 passages of receipt.

### Plasmid construction

Plasmids are listed in S1 Table. Oligonucleotides are found in S2 Table. A low copy arabinose-inducible expression plasmid was constructed by excising the *araC* gene and promoter from pBAD18-Cm using *Cla*I and *Hind*III, and cloning into the same restriction sites of pMPMA3ΔP*lac*. A transcriptional terminator (Part Bba_B0015, parts.igem.org) was subsequently cloned in using the *Sph*I and *Hind*III sites, resulting in the plasmid pMPMA3ΔP*lac* P_BAD_ TT. *invA or sipB* was amplified from SL1344 genomic DNA using oligonucleotides containing a ribosomal binding site and cloned into pMPMA3ΔP*lac* P_BAD_ TT with *Nhe*I and *Sph*I, resulting in the plasmids pP_BAD_-*invA*, and pP_BAD_-*sipB*, respectively. The *sopB-sigE* operon was amplified from the plasmid pSopB^2xHA^ in the same manner, for the resulting plasmid pP_BAD_-*sopB*-2HA.

A cytosolically induced *gfp* reporter was constructed by transcriptionally fusing the promoter of *uhpT* to *gfp* as follows. First, *gfpmut*3.1 was amplified from pFPV25.1 and cloned into the *Xho*I and KpnI sites of pMPMA3ΔP*lac*, resulting in plasmid pMPMA3ΔP*lac*-*gfp*. The *uhpT* promoter region was amplified from SL1344 genomic DNA and cloned into the *Not*I and *BamH*I sites of pMPMA3ΔP*lac*-*gfp*. Finally, part Bba_B0015 (see above) was cloned into *Cla*I and *Xho*I, resulting in the final plasmid pP*uhpT-gfp*. To construct pP*uhpT*-*sopB*-2xHA and the promoterless control plasmid pP_NULL_-*sopB*-2xHA, the *sopB*-2xHA *sigE* operon was amplified from p*sopB*^*2xHA*^ and cloned into *Sph*I and *Hind*III sites of pP*uhpT*-*gfp* or into the *BamH*I and *Xho*I sites of pMPMA3ΔP*lac*-*gfp*, thus replacing the *gfp* gene in each plasmid.

### Bacterial infection of mammalian cells

Cells were passaged 16–18 h prior to infection into 24-well tissue culture treated plates. For C2BBe1 cells, coverslips were pre-coated with collagen type I (BD Biosciences). Bacteria were grown under conditions optimizing T3SS1-dependent invasion [18,19]: a 2 mL overnight culture was subcultured in 10 mL of LB-Miller broth (1:33 dilution, no antibiotics) with shaking at 37°C for 3.5 h. For strains harboring pP_BAD_-*invA*, 0.02% arabinose was added for the duration of the subculture; for pP_BAD_-*sipB*, 0.2% arabinose was added for the duration of the subculture; for Δ*sopB* harboring pP_BAD_-*sopB*-2xHA, 0.2% arabinose was added for the final 1 h of the subculture. Bacteria were pelleted (1 mL, 8,000 x *g*, 2 min) at room-temperature (RT) and re-suspended in an equal volume of Hanks’ Balanced Salt Solution (HBSS, Mediatech). Monolayers were infected at an MOI of ~50 for 10 min at 37°C in 5% CO_2_. Extracellular bacteria were removed by washing with HBSS (x2) and cells were incubated in antibiotic-free CGM until 30 min pi. Cells were then incubated for 1 h in CGM supplemented with L-Histidine (500 μg/mL) and gentamicin (50 μg/mL), followed by CGM supplemented with L-Histidine (500 μg/mL) and gentamicin (10 μg/mL) for the remainder of the infection.

### Gentamicin protection assay

HeLa cells were infected according to the ‘Bacterial Infection of Mammalian cells’ section. At the desired time points, wells were lysed in 0.2% (w/v) sodium deoxycholate to enumerate viable intracellular bacteria.

### Chloroquine resistance assay

HeLa cells were infected according to the ‘Bacterial Infection of Mammalian cells’ section with the following modification: 1 h prior to sample collection, media in replicate wells were replaced with growth media containing gentamicin and histidine, with or without chloroquine (400 μM). Monolayers were lysed in 0.2% (w/v) sodium deoxycholate and serial dilutions plated.

### Proximity ligation assay and image analysis

Proximity Ligation Assays (PLA) were performed using the Red DuoLink *In Situ* PLA Kit (Sigma-Aldrich). Cells were serum starved (0% FBS) 3h prior to and for the duration of the infection. Infected cells were fixed in 2.5% w/v paraformaldehyde (PFA) for10 min at 37°C, washed with PBS and stained with Alexa Fluor 647-conjugated wheat germ agglutinin for 10 min at 37°C (Life Technologies). Cells were washed in PBS, fixed in PFA for 5 min at RT, and blocked and permeabilized in 0.2% (w/v) saponin and 10% (v/v) normal donkey serum in PBS (SS-PBS) for 30 min at RT. The PLA assay was subsequently performed according to the ‘custom solutions’ protocol of the DuoLink *In Situ* PLA kit. Antibodies (goat anti-CSA1, 1:300; KPL and rabbit phospho-Akt Ser473 (D9E), 1:400; Cell Signaling) and PLA probes were diluted in SS-PBS. For HeLa cells, phosphorylated Akt PLA signals were manually quantified from maximum intensity projections assembled from 20 slice stacks. For C2BBe1 cells, signals were quantified using CellProfiler (www.cellprofiler.org) [64] from maximum intensity projections assembled from 15 slice stacks. Our analysis pipeline involved image thresholding followed by nuclei detection in the DAPI channel. A perinuclear area defined by a 50-pixel extension of the identified nucleus object was used to define this region for PLA signal quantification. PLA signals were identified following image thresholding and related to their respective parent nucleus.

### Digitonin permeabilization assay

At the indicated time points, infected HeLa cells were washed three times with KHM buffer (110 mM potassium acetate, 20 mM HEPES, 2 mM MgCl_2_, pH 7.3), and the plasma membrane selectively permeabilized by incubation with digitonin (40 μg/mL in KHM buffer) for 1 min at RT, followed by three washes with KHM buffer. Cells were then incubated for 12 min at RT with rabbit anti-Calnexin (Stressgen, 1:250 in KHM), to label the cytosolic face of the endoplasmic reticulum in permeabilized cells, and goat anti-*Salmonella* CSA1 antibodies (KPL, 1:100 in KHM), to detect cytosolic bacteria. Cells were washed in PBS, fixed in PFA and all host cell membranes were permeabilized with SS-PBS. Antibodies delivered post-digitonin permeabilization were detected with Alexa Fluor 647-conjugated anti-rabbit and Alexa Fluor 568-conjugated anti-goat antibodies. After washes with PBS, SopB^3xFLAG^ and SipA effectors were detected using mouse anti-FLAG M2 (Sigma-Aldrich, 1:250) and mouse anti-SipA (1:50), respectively. Cells were washed again in PBS and incubated with Alexa Fluor 488-conjugated anti-mouse to detect effector bound antibodies and Pacific Blue-conjugated goat anti-*Salmonella* CSA1 to label all intracellular bacteria. Coverslips were then washed sequentially with PBS and distilled water, and mounted on glass slides in a Mowiol solution.

### Immunofluorescence microscopy

Infected cells on coverslips were washed with PBS and fixed in either 2.5% PFA for 10 min at 37°C or 100% ice-cold methanol for 1 min. After three washes with PBS, cells were blocked and permeabilized with SS-PBS for 30 min at RT, incubated in primary antibodies diluted in SS-PBS for 1.5 h at RT, washed sequentially in PBS and saponin-PBS, incubated in Alexa Fluor-conjugated secondary antibodies diluted in SS-PBS for 45 min at RT and washed sequentially in PBS and distilled water. Coverslips were mounted onto glass slides in a Mowiol solution supplemented with 2.5% (w/v) DABCO. Samples were imaged on a Carl Zeiss LSM 710 confocal laser-scanning microscope equipped with a Plan APOCHROMAT 63X/1.4 N.A. objective and assembled into flat maximum-intensity projections using FIJI (NIH) or Zen 2012 SP1 software. Figs were assembled using Adobe Photoshop CC.

#### Antibodies

Primary antibodies used for immunofluorescence studies were as follows: mouse monoclonal anti-SipA (1:50), mouse monoclonal anti-SipB (1:50), mouse anti-FLAG M2 (Sigma-Aldrich, 1:1000), rabbit anti-GFP (Thermofisher, 1:50), mouse anti-HA (Covance, Clone 16B12, 1:400), goat anti-*Salmonella* CSA1 (KPL, 1:300) and rabbit anti-LAMP1 (Difco, 1:500). Alexa Fluor conjugated secondary antibodies were purchased from Life Technologies, diluted 1:1 in sterile glycerol (Sigma-Aldrich) and used at 1:400 dilution. Antibody dilutions specific to certain assays are listed with the appropriate method.

### Immunoblotting

HeLa cells were plated onto 6-well tissue culture treated plates and infected as described above except the monolayers were infected at an MOI of ~100 and serum starved 3h prior to and for the duration of the infection. At the indicated times, monolayers were solubilized in boiling 1.5X Laemmli sample buffer and kept at -20°C until ready for analysis. Protein samples were boiled for 10 min at 95°C, separated by SDS-PAGE (10% v/v bis-acrylamide gels) and transferred to nitrocellulose (0.45μm; Bio-Rad Laboratories) at 100V for 1 h. Membranes were incubated in blocking buffer (5% w/v BSA in Tris-buffered saline/0.1% Tween 20; TBST) for 1 h at RT and incubated overnight at 4°C with primary antibody diluted as recommended by the manufacturer. Membranes were washed three times in TBST and incubated at room temperature for 1 h with appropriate HRP-linked IgG secondary antibody (Cell Signaling Technology; 1:20,000 diluted in blocking buffer). Membranes were washed three times in TBST and developed with SuperSignal West Femto Chemiluminescent Substrate (Thermo Fisher Scientific).

### Mouse infections and processing of gallbladders for cryosectioning

Seven-week old C57BL/6J mice (Jackson Laboratories) were infected by retro orbital i.v. injection with ~500 CFU of *Salmonella* Typhimurium SL1344 SopB^3xFLAG^ or SipA^3xFLAG^ (4 mice per strain). Mice were monitored daily for signs of clinical illness. On days 4 and 5, animals were anesthetized with isofluorane prior to transcardial perfusion with pharmaceutical grade heparin/saline (100 U/mL), followed by perfusion with 4% (w/v) PFA. Gallbladders were harvested and post-fixed in 4% (w/v) PFA for 4 h at RT, washed three times with PBS (30 min each) and cryopreserved overnight in 30% (w/v) sucrose/PBS at 4°C. The following day, gallbladders were incubated in ‘optimal cutting temperature’ compound (OCT; Sakura Finetek) for 15–30 min (RT) and 5 μm sections were prepared on Shanodon Positively Charged Superfrost slides (Thermo Scientific) using a Leica CM3050S Kryostat. Slides were air-dried and stored at -20°C until staining.

### Immunofluorescence staining of cryosections

Cryosectioned slides were equilibrated to room temperature, the OCT layer removed and washed twice with PBS (5–10 min each). To quench free aldehyde groups, samples were incubated for 1 h with 0.3 M Glycine/PBS, rinsed once with PBS, incubated for 10 min with 50 mM NH_4_Cl/PBS and washed again in PBS. Samples were incubated in a humidity chamber for 30 min in block solution (2% donkey serum, 1% BSA, 0.5% Triton X-100, 0.95% Tween-20, PBS pH 7.2) prior to overnight incubation at 4°C with primary antibodies. Slides were washed twice in PBS (10 min each with rocking) and incubated with secondary antibody for 1 h at room temperature. Slides were finally washed twice in PBS and mounted in Prolong Gold Anti-fade reagent with DAPI (Life Technologies). Tile scans of murine gallbladder images were acquired at frame size of 3073 x 3072 pixels with 40X EC Plan-Neofluar 40x/1.30 N.A., 0.6X digital zoom. Antibodies used were as follows: rat anti-LAMP1 1D4B (1:100; Abcam), Alexa Fluor 488-conjugated goat anti-CSA1, Alexa Fluor 568-conjugated mouse anti-FLAG M2 (concentrations determined by user post-conjugation) and Alexa Fluor 647 goat anti-rat (1:400).

### T3SS1 effector secretion assay

SPI1-induced cultures (10 mL) were prepared as detailed in the main Experimental Procedures. 1 mL of culture was pelleted by centrifugation (8,000 x *g*, 2 min, RT), supernatant removed and the pellet resuspended in 250 μL boiling 1.5x Laemmli SDS-PAGE sample buffer, boiled at 95°C for 10 min, and snap-frozen. In parallel, the remaining culture (~ 9 mL) was split between two pre-chilled thick-wall, polycarbonate ultra-centrifuge tubes (Beckman Coulter #355647) on ice and centrifuged at 30,000 x *g* in a MLA-80 rotor in an Optima Max Ultracentrifuge (Beckman Coulter; 20 min, 4°C). Supernatant was carefully removed, filtered through a 0.2 μm PES low-protein binding filter (GE Healthcare) and proteins were precipitated overnight at 4°C in 10% (v/v) trichloroacetic acid. Precipitated sample was divided into pre-chilled 2 mL snap-cap tubes and centrifuged for 20 min at 16,000 x *g* (4°C). Pellets were washed once with ice-cold acetone, centrifuged as above and allowed to dry for 5–10 min in a fume hood. Pellets were resuspended in a total volume of 200 μL pre-heated 1.5X Laemmli sample buffer (95°C), snap frozen and stored at -80°C until use. Aliquots of 10 μL were analyzed by SDS PAGE to determine secretion of relevant effector proteins. Antibodies used for protein detection were as follows: mouse anti-FLAG M2 (1:1000; Sigma-Aldrich) and mouse anti-DnaK (1:20,000; Enzo Lifesciences).

### Cytotoxicity assay

Cytotoxicity assays were performed using the colorimetric CytoTox 96 Non-Radioactive Cytotoxicity assay kit (Promega). HeLa cells were infected as described above except that cells were serum starved (0% FBS) 3 h prior to and for the duration of the infection. Incubations from 0.5 hpi were carried out with the addition of 0.2% BSA to minimize the cytotoxic effects of serum starvation. At 6 hpi, supernatants from sample wells were collected and assayed for lactate dehydrogenase release following manufacturer’s instructions. Absorbance at 492 nm was measured with a Tecan Infinite M200 Pro.

### Live cell microscopy

HeLa cells were plated and infected in black glass-bottom 24-well plates (Grenier BioOne) according to the ‘Bacterial Infection of Mammalian cells’ protocol. When appropriate, cells were treated with either 50 μg/mL AF 488-dextran (Invitrogen) 20h prior to infection or 250 ng/mL propidium iodide (ImmunoChemistry Technologies) 1.5h post-infection. Between 3 and 10 hpi, images were acquired every 5 minutes using a Nikon TiE spinning disc confocal microscope (CSU10 Yokogawa) with Perfect Focus, Cascade II CCD camera (Photometrics), and custom laser launch (Prairie Technologies). All imaging was preformed within a stage-top incubation chamber (Pathology Devices) at 37°C, 75% humidity, and 5% CO_2_. Wells were imaged using a Plan Fluor 40X 0.75 N.A. Ph2 air objective. All post-acquisition image analysis was done using ImageJ software (W.S. Rasband, National Institutes of Health, Bethesda, MD version 2.0.0) and Adobe Photoshop (CS5 v12.1 Adobe).

### Statistical analyses

Unless otherwise stated in the figure legend, data were analyzed for statistical significance by a one-way analysis of variance (ANOVA) with Bonferroni’s post hoc test. A P-value of ≤ 0.05 was considered significant. * = P ≤ 0.05, ** = P ≤0.01, *** = P ≤0.001, ns = not-significant/ P > 0.05.

### Ethical statement

All animal work at Rocky Mountain Laboratories adhered to the U.S. Government Principles and applicable humane and ethical policies in accordance with the Public Health Service (PHS) policy, the Guide for Care and Use of Laboratory Animals and the Animal Welfare Regulations. The Rocky Mountain Laboratories ACUC reviewed and approved this research (ASP# 2014–028).

## Supporting information

S1 Fig Sustained delivery of SipA to epithelial cells by *Salmonella*. Time course of SipA delivery in HeLa cells. Monolayers infected with WT *Salmonella* were PFA fixed and immunostained for bacteria (αCSA) and effector (αSipA). (A) The percentage of effector-positive infected cells was quantified. Means ± SD from 3 independent experiments. (B) Representative confocal images of *Salmonella*-infected HeLa cells with SipA effector staining. Scale bars: 10 μm; inset 2 μm. (C) Analysis of intracellular *Salmonella* populations in SipA-positive cells. Effector-positive cells were classified per the presence or absence of cytosolic bacteria. Means from 3 independent experiments. Statistical significance was analyzed by unpaired Student’s *t*-test. ** P ≤ 0.01, *** P ≤ 0.001. (D) Representative confocal images of HeLa cells infected with WT *Salmonella* subjected to a differential permeabilization assay. Cytosolic (Cyto.) bacteria (red) and the cytosolic tail of calnexin (yellow, permeabilization control) are labeled after digitonin permeabilization. Total bacteria (grey) and SipA (green) were detected post saponin permeabilization. Scale bars: 10 μm; inset 2 μm.
(TIF)

S2 Fig SopB is delivered into gallbladder epithelial cells by hyper-replicating *Salmonella*. (A) Additional confocal images of gallbladder sections at 5 days pi from C57BL/6J mice infected with *Salmonella* expressing SopB^3xFLAG^. Cryosections were immunostained for SopB^3xFLAG^ (αFLAG, grey) and bacteria (αCSA, green). DNA was stained with DAPI (cyan). Arrows indicate effector labeling. Whole gallbladder image (Scale bar: 200 μm) was compiled from tiled images; boxed areas are shown as enlarged overlay (scale bar: 20 μm) and single channel images (Scale bar: 5 μm).
(TIF)

S3 Fig The *uhpT* promoter is induced in cytosolic *Salmonella*. Representative confocal images of HeLa cells infected with WT Salmonella bearing a plasmid borne transcriptional reporter for the hexose phosphate transporter gene, *uhpT* (P*uhpT-gfp*). Cytosolic bacteria are GFP^+^; vacuolar bacteria are GFP^-^. Infected cells were subjected to differential permeabilization at indicated times. Cytosolic bacteria (grey) and the cytosolic tail of calnexin (yellow, permeabilization control) were labeled after digitonin permeabilization. Total bacteria (red) were detected post saponin permeabilization. Scale bars: 10 μm.
(TIF)

S4 Fig Analysis of T3SS1 effector delivery by cytosolic *Salmonella*. (A) The invasion defect of Δ*invA*-*sopB*^3xFLAG^ is complemented by arabinose induction of *invA* in T3SS1^IND^-*sopB*^3xFLAG^. ΔSPI2-*sopB*^3xFLAG^ is invasion competent. Recoverable intracellular CFUs from infected HeLa cells were determined by gentamicin protection assay at 1.5 hpi. Means ± SD of 3 independent experiments. (B-E) Quantification of T3SS1 effector expression by cytosolic *Salmonella* in HeLa (B) and C2BBe1 (C) cells with representative confocal images (D) and (E), respectively. Infected monolayers were methanol fixed at 6 hpi and immunostained for effector (αFLAG or αSipA) and bacteria (αCSA). The percentage of infectedC2BBe1 cells containing >50 bacteria/cell with effector staining was scored. Means ± SD of 3 independent experiments; ns = not significant.C2BBe1 Scale bars: 10 μm; inset 2 μm. (F, I) The invasion defect of Δ*sipB*-*sopB*^3xFLAG^ is complemented by arabinose induction of *sipB* in SipB^IND^-*sopB*^3xFLAG^. Recoverable intracellular CFUs from infected HeLa cells were determined by gentamicin protection assay at 1.5 hpi. Means ± SD of 3 independent experiments. (G, H) Δ*sipB* secretes T3SS1 effectors into broth culture. Western blot for secreted SopB^3xFLAG^ (G) and SipA^3xFLAG^ (H) in culture supernatants and bacterial pellets of the indicated *Salmonella* strains grown in LB-M. DnaK was used to verify equal loading (pellet) and assess bacterial lysis (supernatant). Blots are representative of 2 independent experiments.
(TIF)

S5 Fig Cytosolic WT *Salmonella*, but not SipB^IND^, express detectible levels of the T3SS1 translocator, SipB, in HeLa cells. (A) Representative confocal images of infected HeLa cells stained for SipB. Monolayers were PFA fixed at indicated time points and stained for the translocator (αSipB), bacteria (αCSA) and LAMP1 (αLAMP1). Scale bars: 10 μm; inset 2 μm. (B) The percentage of infected cells with SipB staining was scored. Means ± SD of 3 independent experiments. Statistical significance was analyzed by 2-way ANOVA with Bonferroni’s post-test. *** P ≤ 0.001, ns = not-significant.
(TIF)

S1 Table List of strains and plasmids used in this study. The genotype and source of strains, strain aliases and plasmids used in this study are listed.
(DOCX)

S2 Table List of oligonucleotides used in this study. Oligonucleotides used in this study are listed with their corresponding sequence and associated construct. Restriction sites are underlined and ribosomal binding sites are italicized when present within the oligonucleotide sequence.
(DOCX)

S3 Table Total numbers of cells analyzed in, n, numbers of independent immunofluorescence experiments. The numbers of cells analyzed in each microscopy experiment in this study are listed according to Figure, and includes cell type, time point and strain used in each experiment.
(DOCX)
