# Supplementary figures and images for: A second wave of Salmonella T3SS1 activity prolongs the lifespan of infected epithelial cells
Source: PLoS Pathog. 2017 Apr 20;13(4):e1006354. doi: 10.1371/journal.ppat.1006354 (PMC5413073; doi:10.1371/journal.ppat.1006354)

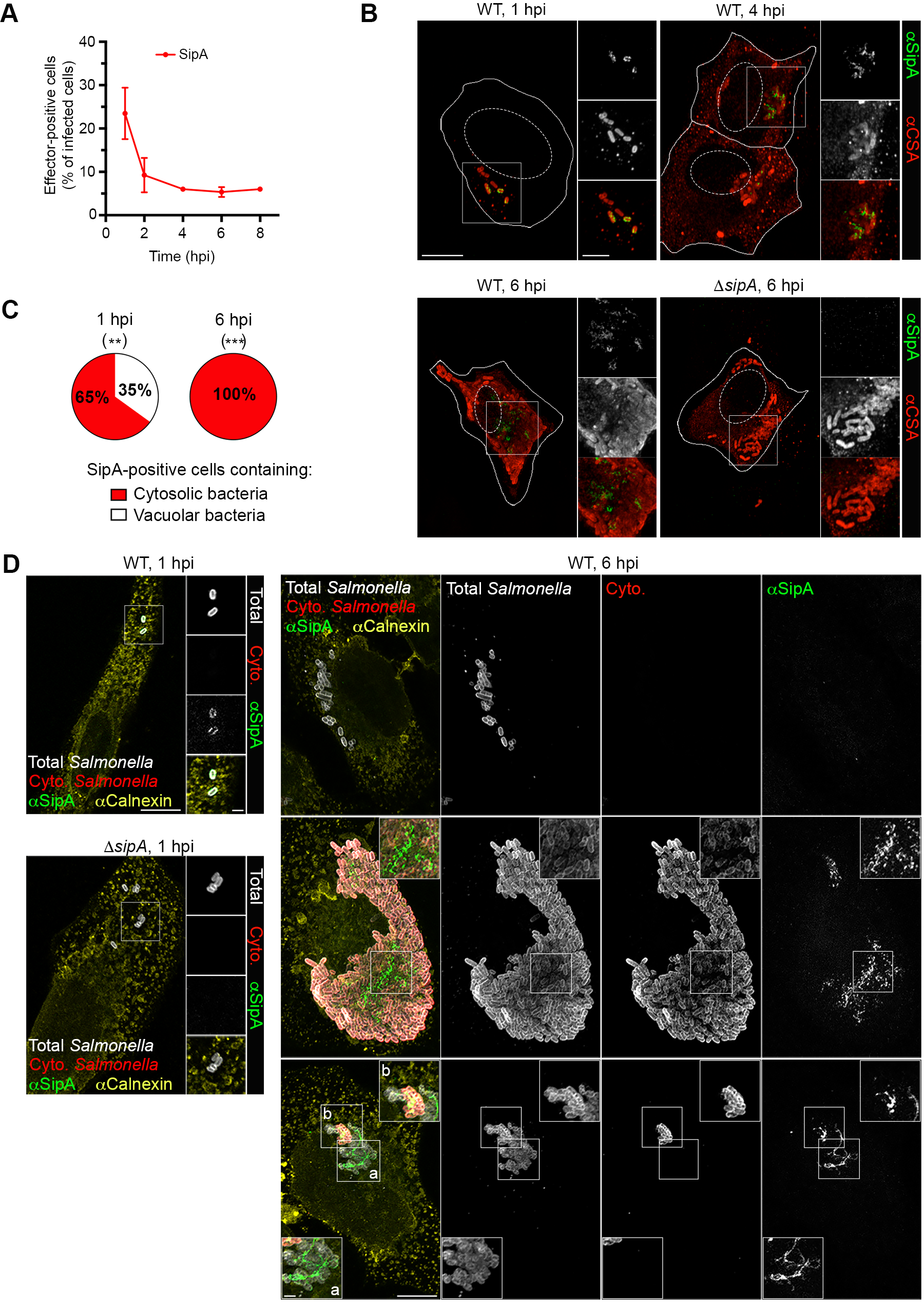

Supplement: S1 Fig — Time course of SipA delivery in HeLa cells. Monolayers infected with WT Salmonella were PFA fixed and immunostained for bacteria (αCSA) and effector (αSipA). (A) The percentage of effector-positive infected cells was quantified. Means ± SD from 3 independent experiments. (B) Representative confocal images of Salmonella-infected HeLa cells with SipA effector staining. Scale bars: 10 μm; inset 2 μm. (C) Analysis of intracellular Salmonella populations in SipA-positive cells. Effector-positive cells were classified per the presence or absence of cytosolic bacteria. Means from 3 independent experiments. Statistical significance was analyzed by unpaired Student’s t-test. ** P ≤ 0.01, *** P ≤ 0.001. (D) Representative confocal images of HeLa cells infected with WT Salmonella subjected to a differential permeabilization assay. Cytosolic (Cyto.) bacteria (red) and the cytosolic tail of calnexin (yellow, permeabilization control) are labeled after digitonin permeabilization. Total bacteria (grey) and SipA (green) were detected post saponin permeabilization. Scale bars: 10 μm; inset 2 μm. (TIF) [file ppat.1006354.s001.tif]

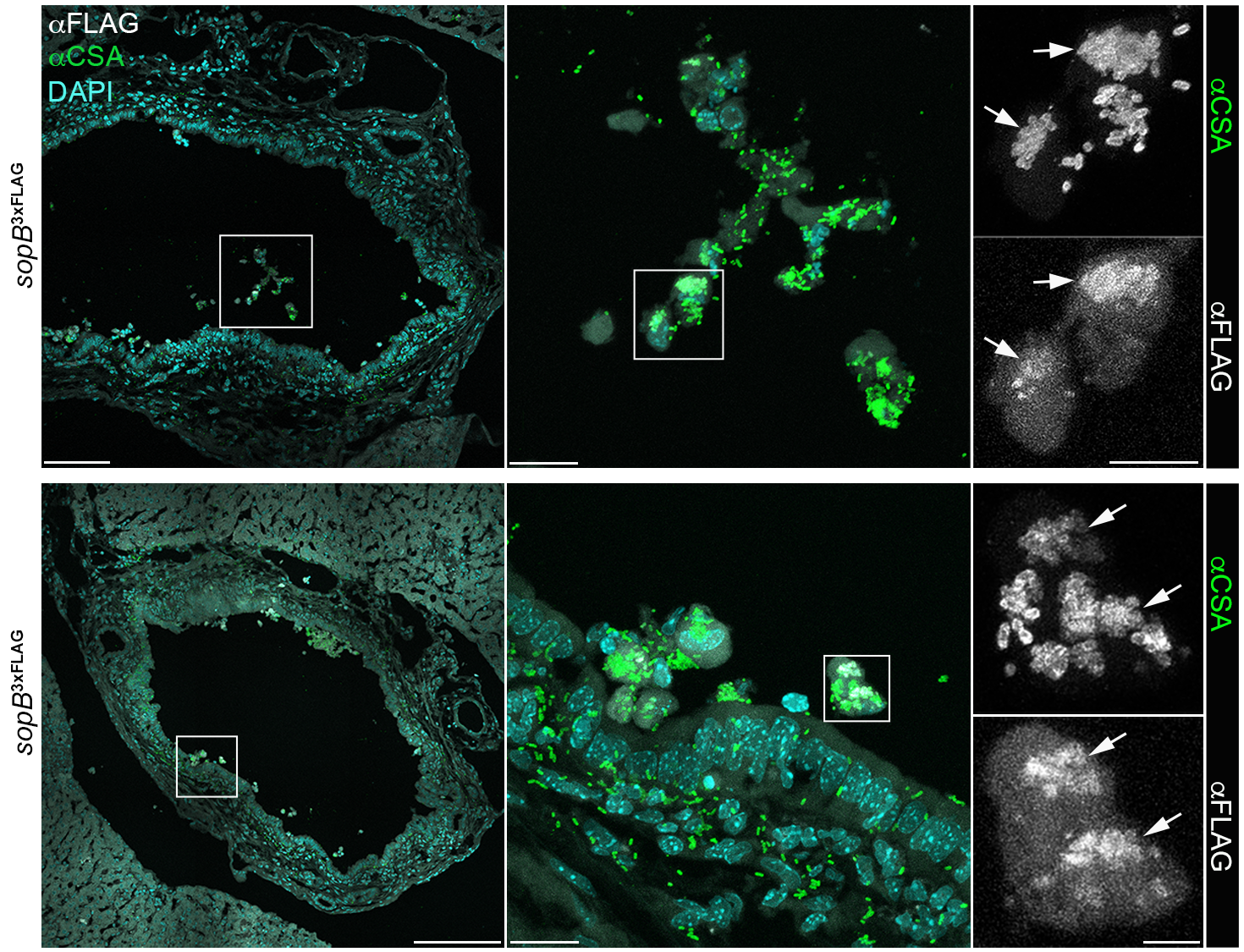

Supplement: S2 Fig — (A) Additional confocal images of gallbladder sections at 5 days pi from C57BL/6J mice infected with Salmonella expressing SopB3xFLAG. Cryosections were immunostained for SopB3xFLAG (αFLAG, grey) and bacteria (αCSA, green). DNA was stained with DAPI (cyan). Arrows indicate effector labeling. Whole gallbladder image (Scale bar: 200 μm) was compiled from tiled images; boxed areas are shown as enlarged overlay (scale bar: 20 μm) and single channel images (Scale bar: 5 μm). (TIF) [file ppat.1006354.s002.tif]

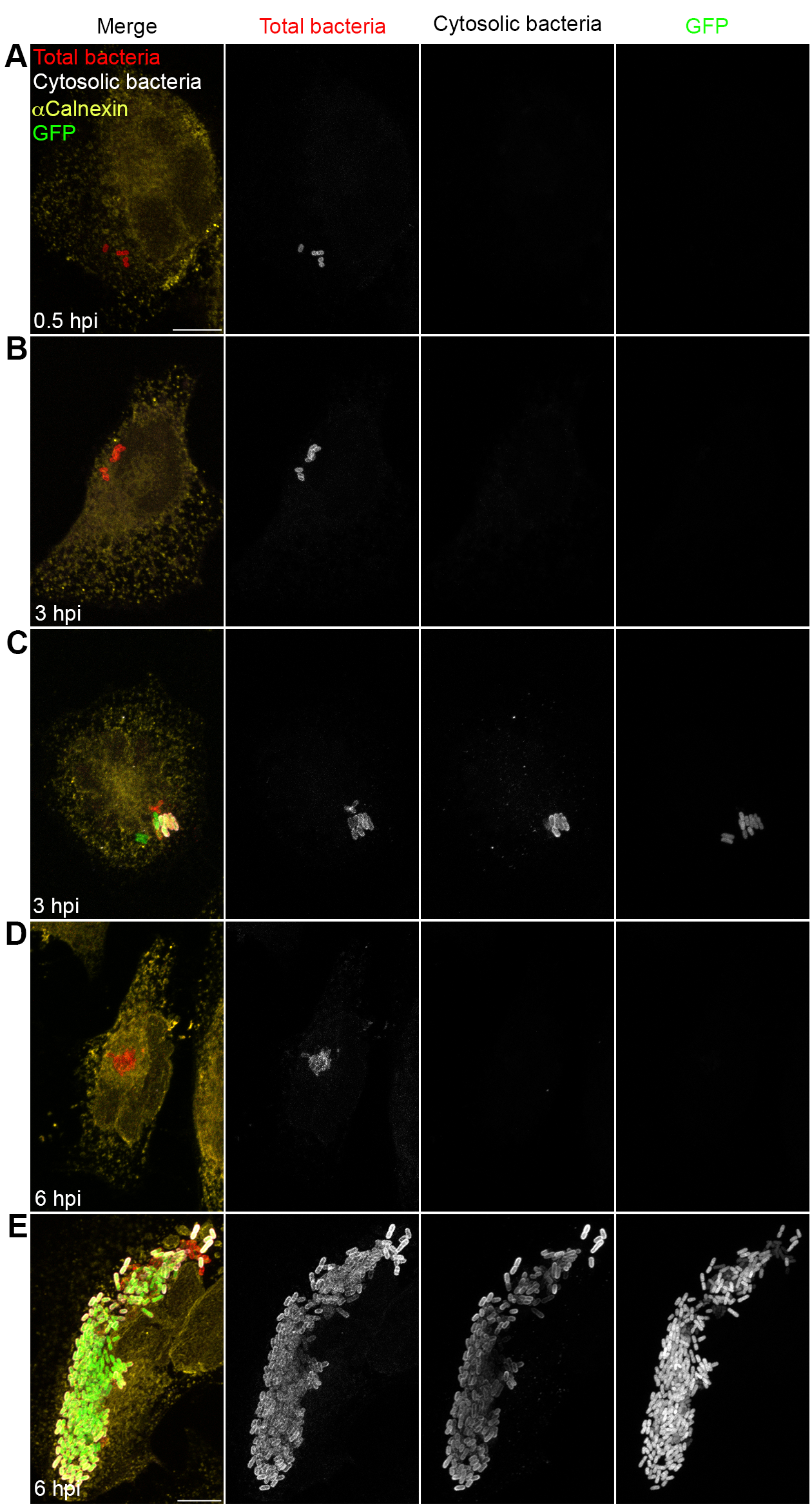

Supplement: S3 Fig — Representative confocal images of HeLa cells infected with WT Salmonella bearing a plasmid borne transcriptional reporter for the hexose phosphate transporter gene, uhpT (PuhpT-gfp). Cytosolic bacteria are GFP+; vacuolar bacteria are GFP-. Infected cells were subjected to differential permeabilization at indicated times. Cytosolic bacteria (grey) and the cytosolic tail of calnexin (yellow, permeabilization control) were labeled after digitonin permeabilization. Total bacteria (red) were detected post saponin permeabilization. Scale bars: 10 μm. (TIF) [file ppat.1006354.s003.tif]

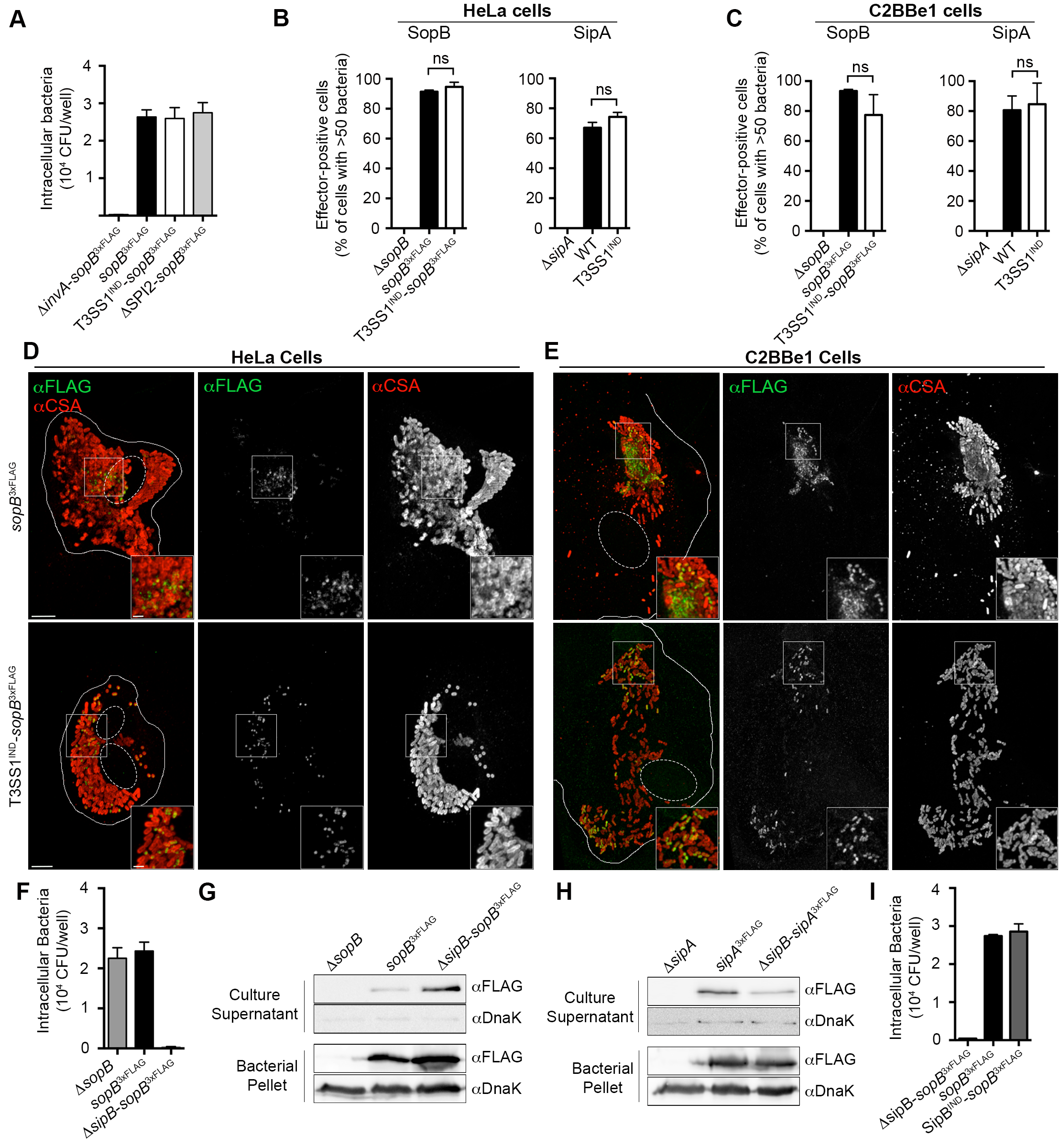

Supplement: S4 Fig — (A) The invasion defect of ΔinvA-sopB3xFLAG is complemented by arabinose induction of invA in T3SS1IND-sopB3xFLAG. ΔSPI2-sopB3xFLAG is invasion competent. Recoverable intracellular CFUs from infected HeLa cells were determined by gentamicin protection assay at 1.5 hpi. Means ± SD of 3 independent experiments. (B-E) Quantification of T3SS1 effector expression by cytosolic Salmonella in HeLa (B) and C2BBe1 (C) cells with representative confocal images (D) and (E), respectively. Infected monolayers were methanol fixed at 6 hpi and immunostained for effector (αFLAG or αSipA) and bacteria (αCSA). The percentage of infectedC2BBe1 cells containing >50 bacteria/cell with effector staining was scored. Means ± SD of 3 independent experiments; ns = not significant.C2BBe1 Scale bars: 10 μm; inset 2 μm. (F, I) The invasion defect of ΔsipB-sopB3xFLAG is complemented by arabinose induction of sipB in SipBIND-sopB3xFLAG. Recoverable intracellular CFUs from infected HeLa cells were determined by gentamicin protection assay at 1.5 hpi. Means ± SD of 3 independent experiments. (G, H) ΔsipB secretes T3SS1 effectors into broth culture. Western blot for secreted SopB3xFLAG (G) and SipA3xFLAG (H) in culture supernatants and bacterial pellets of the indicated Salmonella strains grown in LB-M. DnaK was used to verify equal loading (pellet) and assess bacterial lysis (supernatant). Blots are representative of 2 independent experiments. (TIF) [file ppat.1006354.s004.tif]

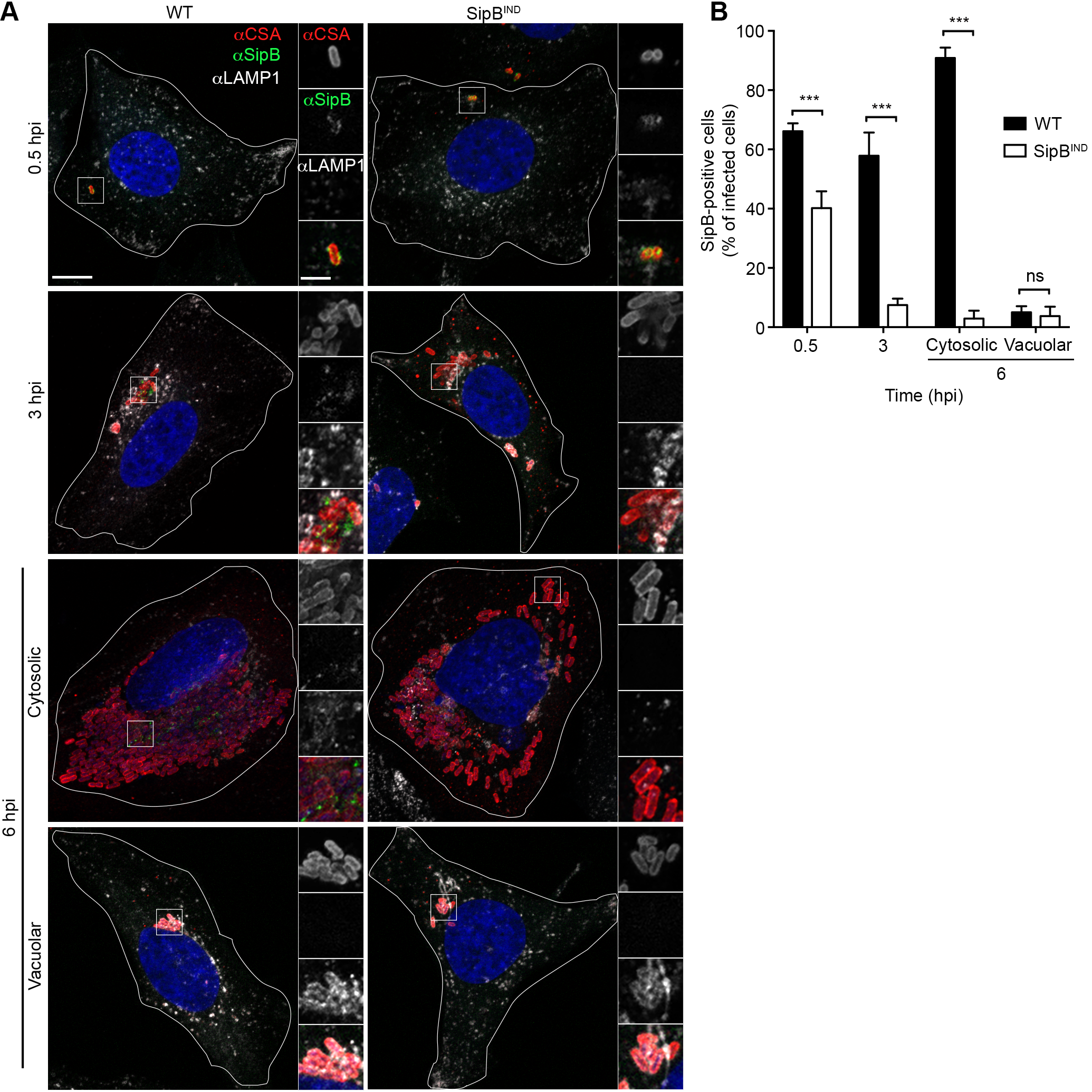

Supplement: S5 Fig — (A) Representative confocal images of infected HeLa cells stained for SipB. Monolayers were PFA fixed at indicated time points and stained for the translocator (αSipB), bacteria (αCSA) and LAMP1 (αLAMP1). Scale bars: 10 μm; inset 2 μm. (B) The percentage of infected cells with SipB staining was scored. Means ± SD of 3 independent experiments. Statistical significance was analyzed by 2-way ANOVA with Bonferroni’s post-test. *** P ≤ 0.001, ns = not-significant. (TIF) [file ppat.1006354.s005.tif]
