# Supplementary material for: A second wave of Salmonella T3SS1 activity prolongs the lifespan of infected epithelial cells
Source: PLoS Pathog. 2017 Apr 20;13(4):e1006354. doi: 10.1371/journal.ppat.1006354 (PMC5413073; doi:10.1371/journal.ppat.1006354)
Supplement: S1 Table — The genotype and source of strains, strain aliases and plasmids used in this study are listed. (DOCX) [file ppat.1006354.s006.docx]

**S1 Table.** List of strains and plasmids used in this study

| Strain | Genotype or Features | Source or Reference |
| --- | --- | --- |
| SL1344 | *hisG46, xyl, rpsL* | Hoiseth and Stocker, 1981 |
| Δ*sopB* | Δ*sopB,* IGR^*^, Δ*sigE*, (a.k.a Δ*sigDE*) | Knodler *et al*., 2006 |
| Δ*sipA* | *sipA*::*kan* | This study |
| Δ*sifA* | *sifA*::*cm* | This study |
| Δ*invA* | *invA*::*kan* | This study |
| Δ*sipB* | *sipB*::*kan* | This study |
| ΔSPI2 | SPI2::*kan* | Knodler *et al.,* 2005 |
| Δ*sopB*, Δ*sifA* | Δ*sopB,* IGR^*^, Δ*sigE* (a.k.a Δ*sigDE*)*, sifA*::*cm* | This study |
| *sopB*^3xFLAG^ | *sopB* chromosomally 3xFLAG tagged | Garcia-del Portillo lab |
| *sipA*^3xFLAG^ | *sipA* chromosomally 3xFLAG tagged | This study |
| Δ*sipB* - *sopB*^3xFLAG^ | *sipB*::*kan*, *sopB* chromosomally 3xFLAG tagged | This study |
| Δ*sipB -* *sipA*^3xFLAG^ | *sipB*::*kan*, *sipA* chromosomally 3xFLAG tagged | This study |
| Δ*invA* - *sopB*^3xFLAG^ | *invA*::*kan*, *sopB* chromosomally 3xFLAG tagged | This study |
| ΔSPI2 - *sopB*^3xFLAG^ | SPI2::*kan, sopB* chromosomally 3xFLAG tagged | This study |
|  |  |  |
|  |  |  |
| **Strain Alias** |  |  |
| T3SS1^IND^ | *invA*::*kan* carrying P_BAD_-*invA* | This study |
| T3SS1^IND^- *sopB*^3xFLAG^ | *invA*::*kan,* *sopB*^3xFLAG^ carrying P_BAD_-*invA* | This study |
| SipB^IND^ | *sipB*::*kan* carrying P_BAD_-*sipB* | This study |
| SipB^IND^- *sopB*^3xFLAG^ | *sipB*::*kan*, *sopB*^3xFLAG^ carrying P_BAD_-*sipB*, | This study |
| SipB^IND^ - *sipA*^3xFLAG^ | *sipB*::*kan*, *sipA*^3xFLAG^ carrying P_BAD_-*sipB*, | This study |
|  |  |  |
|  |  |  |
| **Plasmid** |  |  |
| PKD4 | FRT-*kan-*FRT template | Datsenko and Wanner, 2000 |
| PKD3 | FRT-*cm-*FRT template | Datsenko and Wanner, 2000 |
| PKD46 | λ red expression plasmid | Datsenko and Wanner, 2000 |
| pSUB11 | 3xFLAG FRT-*kan*-FRT template | Uzzau, *et al*., 2001 |
| pSopB^WT^ | P*sopB* driven *sopB* and *sigE* (a.k.a. pWSKDE) | Knodler *et al*., 2009 |
| pSopB^2xHA^ | P*sopB* driven *sopB-*2xHA and *sigE* (a.k.a. pWSKDE-2xHA) | Knodler *et al*., 2009 |
| pSopB^C460S^ | P*sopB* driven *sopB* (catalytically inactive) and *sigE* (a.k.a. pWSKDE^C460S^) | This study |
| pMPMA3ΔP*lac* | P15A *ori*, pMPMA3 with *lac* promoter removed | Ibarra *et al*., 2010 |
| pBAD18-*Cm* | pBR322 *ori*, arabinose inducible expression plasmid | Guzman *et al*., 1995 |
| pFPV25.1 | ColE1 *ori*, P*rpsM* driven *gfp* | Valdivia and Falkow, 1996 |
| pMPMA3ΔPlac P_BAD_ TT | P15A *ori*, arabinose inducible expression plasmid | This study |
| pMPMA3ΔPlac-*gfp* | P15A *ori*, promoterless *gfp* | This study |
| pP*prgH*-*gfp*[LVA] | P15A *ori*, *prgH* transcriptional *gfp* reporter | Ibarra *et al*., 2010 |
| pP*uhpT*-*gfp* | P15A *ori*, *uhpT* trancriptional *gfp* reporter | This study |
| pP*uhpT*-*sopB*^2xHA^ | P15A *ori*, P*uhpT* driven *sopB-*2HA and *sigE*, G6P^**^-inducible | This study |
| pP_BAD_-*invA* | P15A *ori*, P_BAD_ driven *invA*, arabinose-inducible | This study |
| pP_BAD_-*sipB* | P15A *ori*, P_BAD_ driven *sipB,* arabinose-inducible | This study |
| pP_BAD_-*sopB*^2xHA^ | P15A *ori*, P_BAD_ driven *sopB-*2HA and *sigE,* arabinose-inducible | This study |
| pP_NULL_-*sopB*^2xHA^ | P15A *ori*, Promoterless *sopB-*2HA and *sigE* | This study |
| pRFP | ColE1 *ori*, P*rpsM* driven *rfp*, causes intracellular replication defect | Knodler *et al*., 2005 |

*IGR: intergenic region

**G6P: glucose-6-phosphate
