# Supplementary material for: A second wave of Salmonella T3SS1 activity prolongs the lifespan of infected epithelial cells
Source: PLoS Pathog. 2017 Apr 20;13(4):e1006354. doi: 10.1371/journal.ppat.1006354 (PMC5413073; doi:10.1371/journal.ppat.1006354)
Supplement: S2 Table — Oligonucleotides used in this study are listed with their corresponding sequence and associated construct. Restriction sites are underlined and ribosomal binding sites are italicized when present within the oligonucleotide sequence. (DOCX) [file ppat.1006354.s007.docx]

**S2 Table.** List of oligonucleotides used in this study

| Oligonucleotide | Sequence (5’-3’)* | Associated strain/plasmid |
| --- | --- | --- |
| *invA* KO F | CTTCTCTATTGTCACCGTGGTCCAGTTTATCGTTATTACCTGTAGGCTGGAGCTGCTTCG | Δ*invA* |
| *invA* KO R | GACATCGACAGACGTAAGGAGGACAAGATCTTTATGTGCCATATGAATATCCTCCTTAG |  |
| *sipA* KO F | AACAGAAGAGGATATTAATAATGGTTACAAGTGTAAGGTGTAGGCTAGAGCTGCTTCG | Δ*sipA* |
| *sipA* KO R | CTTTCCCGGTTAATTAACGCTGCATGTGCAAGCCATCAACGCATATGAATATCCTCCTTAG |  |
| *sipB* KO F | AGCACAGTGAACAAGAAAAGGAATAATTATGGTAAATGACGCTGTAGGCTGGAGCTGCTTCG | Δ*sipB* |
| *sipB* KO R | ATTTAAATAAGCGGCGGGATTTATTCCCACATTACTAATTAACATATGAATATCCTCCTTAG |  |
| *sifA* KO F | TCCAGTATAAGTGAGATTAATATGCCGATTACTATAGGGTGTAGGCTGGAGCTGCTTCG | Δ*sifA* |
| *sifA* KO R | CCCTGAACGTGACGTCTGAGAAAGCGTCGTCTGATTTTACATATGAATATCCTCCTTAG |  |
| *sipA* 3xFLAG F | GTTATTACTACCGTTGATGGCTTGCACATGCAGCGTGACTACAAAGACCATGACGC | *sipA*::3xFLAG |
| *sipA* 3xFLAG R | CTTCAATATCCATATTCATCGCATCTTTCCCGGTTACATATGAATATCCTCCTTAG |  |
| B0015 *Sph*I F | NNNGCATGCCCAGGCATCAAATAAAACGAAA | pMPMA3ΔPlac P_BAD_ TT |
| B0015 *Hind*III R | NNNAAGCTTTATAAACGCAGAAAGGCCC | pMPMA3ΔPlac P_BAD_ TT |
| *invA Nhe*I RBS F | NNNGCTAGC*AGGAGGAATTAACC*GTGCTGCTTTCTCTACTTAACAG | P_BAD_-*invA* |
| *invA Sph*I R | NNNNNNGCATGCTTATATTGTTTTTATAACATTCACTGAC | P_BAD_-*invA* |
| *sipB Nhe*I RBS F | NNNGCTAGC*AGGAGGAATTAACC*ATGGTAAATGACGCAAGTAGC | P_BAD_-*sipB* |
| *sipB Sph*I R | NNNNNNGCATGCTTATGCGCGACTCTGGCGCAG | P_BAD_-*sipB* |
| *sopB Nhe*I RBS F | NNNGCTAGC*AGGAGGAATTAACC*ATGCAAATACAGAGCTTCTATCA | P_BAD_-*sopB*-2HA |
| *sigE Sph*I R | NNNNNNGCATGCTTATGCATAATGCTCTTTCAATTG | P_BAD_-*sopB*-2HA |
| *gfp Xho*I F | NNNCTCGAGATGCGTAAAGGAGAAGAACTT | pMPMA3ΔPlac-*gfp* |
| *gfp Kpn*I R | NNNNNNGGTACCTTAAGCTACTAAAGCGTAGT | pMPMA3ΔPlac-*gfp* |
| P*uhpT*­ ­*Not*I F | TAGCTGTGGCGGCCGCAGACCCAGAAGCGTG | P*uhpT*-*gfp* |
| P*uhpT*­ pMPMA3 *BamH*I R | GGTACCCGGGGATCCGGATTACTCCTGAGCTAATTTTTAT | P*uhpT*-*gfp* |
| B0015 *Cla*I F | NNNNATCGATCCAGGCATCAAATAAAACGAAA | P*uhpT*-*gfp* |
| B0015 *Xho*I R | NNNCTCGAGATAAACGCAGAAAGGCCC | P*uhpT*-*gfp* |
| *sopB Sph*I F | TCGGTAAGCATGCAAATACAGAGCTTCTATCTATC | P*uhpT*-*sopB-*2xHA |
| *sigE Hind*III R | GCGGTAAGCTTTTATGCATAATGCTCTTTCAATTG | P*uhpT*-*sopB-*2xHA |

*Note: Restriction sites are underlined and ribosomal binding sites are italicized.
