# Supplementary material for: A second wave of Salmonella T3SS1 activity prolongs the lifespan of infected epithelial cells
Source: PLoS Pathog. 2017 Apr 20;13(4):e1006354. doi: 10.1371/journal.ppat.1006354 (PMC5413073; doi:10.1371/journal.ppat.1006354)
Supplement: S3 Table — The numbers of cells analyzed in each microscopy experiment in this study are listed according to Figure, and includes cell type, time point and strain used in each experiment. (DOCX) [file ppat.1006354.s008.docx]

**SUPPORTING TABLE**

**S3 Table.** Total numbers of cells analyzed in, n, numbers of independent immunofluorescence experiments.

| **Figure** | **Cell Type** | **Time point** | **Strain** | **Total cells analyzed** |
| --- | --- | --- | --- | --- |
| 1B | HeLa | all time points | WT | 150 (n=3) |
| 1D | HeLa | 1 hpi | WT | 92 (n=3) |
|  |  |  | Δ*sopB*/p*sopB*^C460S^ | 92 (n=3) |
|  |  |  | Δ*sopB*/p*sopB*^WT^ | 92 (n=3) |
|  |  | 3 hpi | WT | 92 (n=3) |
|  |  |  | Δ*sopB*/p*sopB*^C460S^ | 92 (n=3) |
|  |  |  | Δ*sopB*/p*sopB*^WT^ | 92 (n=3) |
|  |  | 6 hpi (<20 bac/cell) | WT | 92 (n=3) |
|  |  |  | Δ*sopB*/p*sopB*^C460S^ | 92 (n=3) |
|  |  |  | Δ*sopB*/p*sopB*^WT^ | 92 (n=3) |
|  |  | 6 hpi (>20 bac/cell) | WT | 92 (n=3) |
|  |  |  | Δ*sopB*/p*sopB*^C460S^ | 92 (n=3) |
|  |  |  | Δ*sopB*/p*sopB*^WT^ | 92 (n=3) |
| 1E | C2BBe1 | 6 hpi (<20 bac/cell) | WT | 45 (n=3) |
|  |  |  | Δ*sopB*/p*sopB*^C460S^ | 41 (n=3) |
|  |  |  | Δ*sopB*/p*sopB*^WT^ | 53 (n=3) |
|  |  | 6 hpi (>20 bac/cell) | WT | 67 (n=3) |
|  |  |  | Δ*sopB*/p*sopB*^C460S^ | 59 (n=3) |
|  |  |  | Δ*sopB*/p*sopB*^WT^ | 56 (n=3) |
|  |  |  |  |  |
| 2A | HeLa | all time points | *sopB*^3xFLAG^ | 150 (n=3) |
| 2C | HeLa | 1 hpi | *sopB*^3xFLAG^ | 150 (n=3) |
|  |  | 6 hpi | *sopB*^3xFLAG^ | 90 (n=3) |
|  |  |  |  |  |
| 5E and 5G | HeLa | 3-10 hpi | WT/pP*uhpT-gfp* | 16 (n=6) |
|  |  | 3-10 hpi | Δ*sopB*/pP*uhpT-gfp* | 17 (n=6) |
| 5F | HeLa | 3-10 hpi | WT/pP*uhpT-gfp* | 40 (n=11) |
|  |  |  | Δ*sopB*/pP*uhpT-gfp* | 25 (n=9) |
|  |  |  | WT/pRFP | 19 (n=5) |
|  |  |  |  |  |
| 7E | HeLa | 6 hpi (SopB) | *sopB*^3XFLAG^ | 150 (n=3) |
|  |  |  | T3SS1^IND^- *sopB*^3XFLAG^ | 150 (n=3) |
|  |  |  | ΔSPI2- *sopB*^3XFLAG^ | 150 (n=3) |
|  |  | 6 hpi (SipA) | WT | 150 (n=3) |
|  |  |  | T3SS1^IND^ | 150 (n=3) |
|  |  |  | ΔSPI2 | 150 (n=3) |
| 7F | C2BBe1 | 6 hpi (SopB) | SopB^3XFLAG^ | 150 (n=3) |
|  |  |  | T3SS1^IND^- *sopB*^3XFLAG^ | 150 (n=3) |
|  |  |  | ΔSPI2- *sopB*^3XFLAG^ | 150 (n=3) |
|  |  | 6 hpi (SipA) | WT | 150 (n=3) |
|  |  |  | T3SS1^IND^ | 150 (n=3) |
|  |  |  | ΔSPI2 | 150 (n=3) |
|  |  |  |  |  |
| 8C | HeLa | 6 hpi (SopB) | *sopB*^3XFLAG^ | 143 (n=3) |
|  |  |  | SipB^IND^- *sopB*^3XFLAG^ | 96 (n=3) |
|  |  | 6 hpi (SipA) | *sipA*^3XFLAG^ | 150 (n=3) |
|  |  |  | SipB^IND^- *sipA*^3XFLAG^ | 73 (n=3) |
| 8E | C2BBe1 | 6 hpi (SopB) | *sopB*^3XFLAG^ | 150 (n=3) |
|  |  |  | SipB^IND^- *sopB*^3XFLAG^ | 134 (n=3) |
|  |  | 6 hpi (SipA) | *sipA*^3XFLAG^ | 150 (n=3) |
|  |  |  | SipB^IND^- *sipA*^3XFLAG^ | 121 (n=3) |
|  |  |  |  |  |
| S1A | HeLa | all time points | WT | 150 (n=3) |
| S1C | HeLa | 1 hpi | WT | 150 (n=3) |
|  |  | 6 hpi | WT | 90 (n=3) |
|  |  |  |  |  |
| S4B | HeLa | 6 hpi (SopB) | Δ*sopB* | 130 (n=3) |
|  |  |  | *sopB*^3XFLAG^ | 150 (n=3) |
|  |  |  | T3SS1^IND^- *sopB*^3XFLAG^ | 150 (n=3) |
|  |  | 6 hpi (SipA) | Δ*sipA* | 71 (n=3) |
|  |  |  | WT | 150 (n=3) |
|  |  |  | T3SS1^IND^ | 118 (n=3) |
|  |  |  |  |  |
| S4C | C2BBe1 | 6 hpi (SopB) | Δ*sopB* | 150 (n=3) |
|  |  |  | *sopB*^3XFLAG^ | 150 (n=3) |
|  |  |  | T3SS1^IND^- *sopB*^3XFLAG^ | 150 (n=3) |
|  |  | 6 hpi (SipA) | Δ*sipA* | 150 (n=3) |
|  |  |  | WT | 150 (n=3) |
|  |  |  | T3SS1^IND^ | 150 (n=3) |
|  |  |  |  |  |
| S5B | HeLa | 0.5 hpi | WT | 199 (n=3) |
|  |  |  | SipB^IND^ | 208(n=3) |
|  |  | 3 hpi | WT | 203(n=3) |
|  |  |  | SipB^IND^ | 175 (n=3) |
|  |  | 6 hpi (Cytosolic) | WT | 138 (n=3) |
|  |  |  | SipB^IND^ | 141 (n=3) |
|  |  | 6 hpi (Vacuolar) | WT | 158 (n=3) |
|  |  |  | SipB^IND^ | 156 (n=3) |
